# Transfer Learning with Uncertainty Quantification: Random Effect Calibration of Source to Target (RECaST)

**Published:** 2024

**Authors:** Jimmy Hickey, Jonathan P. Williams, Emily C. Hector

**Affiliations:** Department of Statistics, North Carolina State University; Department of Statistics, North Carolina State University, Centre for Advanced Study, Norwegian Academy of Science and Letters; Department of Statistics, North Carolina State University

**Keywords:** Bayesian transfer learning, Electronic health records, Informative Bayesian prior, Model calibration

## Abstract

Transfer learning uses a data model, trained to make predictions or inferences on data from one population, to make reliable predictions or inferences on data from another population. Most existing transfer learning approaches are based on fine-tuning pre-trained neural network models, and fail to provide crucial uncertainty quantification. We develop a statistical framework for model predictions based on transfer learning, called *RECaST*. The primary mechanism is a Cauchy random effect that recalibrates a source model to a target population; we mathematically and empirically demonstrate the validity of our RECaST approach for transfer learning between linear models, in the sense that prediction sets will achieve their nominal stated coverage, and we numerically illustrate the method’s robustness to asymptotic approximations for nonlinear models. Whereas many existing techniques are built on particular source models, RECaST is agnostic to the choice of source model, and does not require access to source data. For example, our RECaST transfer learning approach can be applied to a continuous or discrete data model with linear or logistic regression, deep neural network architectures, etc. Furthermore, RECaST provides uncertainty quantification for predictions, which is mostly absent in the literature. We examine our method’s performance in a simulation study and in an application to real hospital data.

## 1 Introduction

The use of artificial intelligence and machine learning (ML) is frequently limited in practice by a shortage of available training data and insufficient computational resources. To address these difficulties, transfer learning has developed as a powerful idea for leveraging the resources at leading institutions such as research hospitals (e.g., institutions having high quality data, exceptional research clinicians, high performance computing environments, etc.) to facilitate implementation of ML technologies in resource scarce settings such as small or rural hospitals. Developments in transfer learning methodologies are necessary to overcome resource allocation inequities, and they will likely drive the next decade of innovation in ML technologies.

Transfer learning consists broadly of two elements. The first is one or more *target* population(s) of interest that are associated with data sets for which there are resource limitations preventing the training of sophisticated models (e.g., a small hospital). The second is a *source* population (or populations) that is separate but in some way related to the target population. The source is associated with extensive data and/or resources for training sophisticated ML models. The premise of transfer learning is to use trained source models to aid in the training of target models. The source and targets are each composed of two components: a *domain*, denoted 𝒟, and a *task*, denoted 𝒯. A domain 𝒟:={𝒳,P(x)} consists of a feature space 𝒳 and a marginal probability distribution P(x) over x∈𝒳. A task 𝒯:={𝒴,P(y∣x)} is composed of a label space 𝒴 and a conditional distribution P(y∣x) over y∈𝒴 given x∈𝒳. Traditional ML is described by the source and target sharing the same domain, ${𝒟}_{S}={𝒟}_{T}$, and sharing the same task, ${𝒯}_{S}={𝒯}_{T}$. Transfer learning problems arise when the source and target domains and/or the source and target tasks are similar but different. We propose a new Bayesian transfer learning framework termed Random Effect Calibration of Source to Target (RECaST) for source and target data sets that share the same outcome space but possibly have different feature-to-outcome mappings.

### 1.1 Our Contributions

Early efforts in transfer learning focused on using labeled data to learn about unlabeled data from the *same* population (see Joachims and Vapnik (2009) for examples). In contrast, modern transfer learning methods explore how knowledge from one source domain can be applied to a *different* target domain. In this spirit, we consider transfer learning in the supervised learning problem that dominates ML applications. Our proposed method uses information from the source and target features and labels to build a predictive model that can be applied to obtain predictions of labels for new target data features of interest. The use of target labels is common across transfer learning and is sometimes referred to as *inductive* transfer learning (Pan and Yang, 2010). For example, a method is proposed in Donahue et al. (2014) to generalize a model built on ImageNet data for use on different labeled target data sets. A neural network is fine-tuned in Shao et al. (2019) to identify and classify machine faults. In Goussies et al. (2014), a decision forest is proposed that uses mixed information gain and label propagation to improve image and gesture recognition in the target domain.

RECaST is a Bayesian framework applied to the transfer learning setting where the feature-to-outcome mappings P(y∣x) may differ between the source and target. For example, source and target hospitals might record largely the same patient data features, but nuances in clinician practices/procedures, inconsistencies in data quality, population disparities, etc. may affect the suitability of using the source mapping as the target mapping. RECaST uses an estimated source model in tandem with the target data to estimate the distributions of a random effect that links the two domains. It then uses the estimated posterior distribution of the random effect parent parameters to construct a posterior predictive distribution of the outcome variable associated with a new target feature. The posterior predictive credible sets obtained through RECaST deliver critical quantification of prediction uncertainty that is lacking in most existing frameworks.

Two primary advantages of RECaST are its scalability, requiring estimation of only 2–3 parameters with no tuning parameters, and that it is agnostic to the source model specification. Importantly, RECaST only requires the source model and parameter estimates, not the source data itself; this is an immense benefit to applications with privacy concerns, such as with medical data. Further, we show that RECaST is asymptotically valid in the canonical case of distinct source and target Gaussian linear models, in that the coverage of prediction sets are guaranteed to asymptotically achieve their stated nominal level of significance.

To evaluate our proposed RECaST approach, we design synthetic simulation studies with both continuous and binary response data reflecting a variety of difficulty levels of transfer learning problems. Next, we investigate the performance of RECaST in real data simulations that arise by permuting real patient data from the multi-center eICU Collaborative Research Database (Pollard et al., 2018). A variety of both point-valued and set-valued prediction metrics are considered, including the empirical coverage of prediction sets. The performance of RECaST is compared to other state-of-the-art transfer learning approaches, including other source-free methods that do not require the source data while learning the target model. These include freeze-unfreeze approaches that are popular for neural networks, as well as a method based on adapting a random forest built on the source data to the target data (Gu et al., 2022). In some cases, it may be possible to have access to both the source and target data. As such we also compare RECaST to methods that require both data sets during training. These include an adversarial learning method (Shen et al., 2018), a method based on penalized GLMs (Tian and Feng, 2022), and a popular weighting approach used on clinical data (Wiens et al., 2014).

The remainder of our paper is organized as follows. We discuss related works in transfer learning in Section 2. In Section 3, we develop the theoretical basis for RECaST and its uncertainty quantification. We then develop Bayesian parameter estimation and prediction procedures in both the continuous and binary response cases in Sections 4 and 5, respectively. We conduct extensive simulation studies in Section 6 by exploring transfer learning problems of a range of difficulties. Section 7 considers a real data analysis for predicting shock in ICU data. Section 8 concludes. Proofs and computational details are provided in the Appendix. Throughout the paper we keep to the convention in the statistical literature of using (·) for innermost grouping followed by {·} and finally [·]. Thus, an expression with many nested parentheses respects the ordering [{(·)}].

## 2 Related Work

General survey papers on transfer learning topics include Pan and Yang (2010); Lu et al. (2015); Weiss et al. (2016); Dube et al. (2020). For hospital disease risk and mortality prediction problems, Wiens et al. (2014), Gong et al. (2015), and Desautels et al. (2017) propose transfer learning approaches based on training algorithms using a learned weighted combination of source and target patient observations. These methods learn many parameters and require access to the source data. RECaST may at first glance appear similar to density ratio estimation, a common approach to transfer learning. Density ratio transfer learning methods, such as the one described in Stojanov et al., seek to learn the relationship between the source and target data via a ratio of their densities; however, these methods require joint access to the source and target data – a limitation avoided by RECaST. In Paul et al. (2016), Raghu et al. (2019), and Ahishakiye et al. (2021), approaches are considered to improve classification accuracy for medical imaging tasks using pre-trained deep neural networks (DNNs) on the ImageNet database (Deng et al., 2009). In the context of ICU patient monitoring, in Shickel et al. (2021) a data augmenting-based transfer learning approach is built for fitting a single-layer recurrent neural network trained on electronic health records (EHR) and wearable device data. Their model is limited in scope to only predicting the binary response of successful versus unsuccessful discharge from a hospital. Implemented in Gao and Cui (2021) is a transfer learning strategy for precision medicine in survival analysis with clinical omics data sets via freezing layers of a pre-trained Cox neural network. Developed in Lee et al. (2012) is a method using support vector machines to predict surgical mortality. Another approach, from Gu and Duan (2022), is to generate additional synthetic target data from a source data set and adjust for heterogeneity in order to predict extreme obesity from medical records and genomics data. An example of low-dimensional representation transfer learning is given in Maurer et al. (2015), and *online* transfer learning is considered in Zhao et al. (2014); Wu et al. (2017). These applied methods are useful in modeling specific pieces of EHR data for prediction, but lack uncertainty quantification. Additionally, some require the learning of many parameters and access to the entire source data set.

Bayesian transfer learning adaptations include Baxter (1998), Raina et al. (2006), Wohlert et al. (2018), Bueno et al. (2020), Chandra and Kapoor (2020), Yang et al. (2020), Zhou et al. (2020), Abba et al. (2023); all except Baxter (1998) and Raina et al. (2006) are based on priors specified from neural network models fitted to source data sets. A posterior distribution fitted to a source DNN model is used as a prior on the parameters for the target task in Wohlert et al. (2018), and the model is trained using mean field variational Bayes (for a reference on variational Bayes, see Zhang et al., 2017). Boosting approaches to transfer learning are considered by Freund and Schapire (1999), Dai et al. (2007), and Desautels et al. (2017). In Abba et al. (2023), a penalized complexity prior between the source and target tasks is considered. While uncertainty quantification for predictions in transfer learning applications is mostly absent in the literature, approximate inference from Bayesian neural networks is used in Roy et al. (2022) to quantify uncertainty in parameter estimates and predictions to account for misaligned feature distributions. This approach, referred to as U-SFAN, is related to our RECaST framework in that it is source-free, but it requires that the source model is a neural network. Another difference is that U-SFAN focuses on using uncertainty in the source domain to guide uncertainty quantification in the target model, whereas RECaST provides uncertainty quantification directly based on the target predictions themselves.

It is important to note the difference between source-free transfer learning methods and “source-free domain adaption” (SFDA) methods: RECaST aims to use labels in the target domain in tandem with a model built in the source domain to learn about the target domain. SFDA methods, in contrast, have neither access to source data nor target labels, and often proceed by learning pseudo-labels for the target data. A comprehensive survey of SFDA approaches is given in Li et al. (2024). These surveyed strategies are predominantly non-model based, purely empirical, and lack a unified underlying framework. Moreover, those that focus on fine-tuning pre-trained neural network models on a target data set require the source model to be a neural network, and often fail to provide crucial uncertainty quantification.

Recently, there have been efforts to investigate theoretical properties of transfer learning approaches. For instance, a learning method based on LASSO for high-dimensional penalized linear regression is considered in Li et al. (2022), while diminishing the effect of *negative transfer*. Negative transfer occurs when including source data negatively impacts the performance on target data. In a similar setting, asymptotically valid confidence intervals for generalized linear model (GLM) parameters in high-dimensional transfer learning problems are established in Tian and Feng (2022). This technique is adapted to a more complicated federated transfer learning setting in Li et al. (2023). A parameter is defined in Cai and Wei (2021) to calculate an “effective sample size” to quantify total amount of information that can be transferred when the source and target conditional distributions differ. This approach is extended in Reeve et al. (2021), where assumptions are relaxed on the relationship between the source and target conditional distributions. Hector and Martin (2022) propose and study the inferential properties of an information-driven shrinkage estimator that is robust to heterogeneity between source and target feature-to-label mappings but assumes this mapping is of the same parametric form. These methods offer more mathematically rigorous motivations, but are restrictive in their modeling options. Such restrictions are eliminated in our proposed framework.

## 3 RECaST Framework

Our transfer learning problem is defined by the following four assumptions: (i) there is a well-developed structural component of the prediction model for the source domain denoted by $f\left(\theta_{S},x_{S}\right)$ which represents the relationship between the features and parameters; (ii) there exist ample source data for estimating the parameter(s) $\theta_{S}$; (iii) ${𝒳}_{S}={𝒳}_{T}$, and the structural component of the target prediction model, denoted by $g\left(\theta_{T},x_{T}\right)$, is believed to be *similar* to $f\left(\theta_{S},x_{T}\right)$; and (iv) there does not exist sufficient target data for reliably estimating the parameter(s) $\theta_{T}$. We hereafter refer to $f\left(\theta_{S},x_{S}\right)$ and $g\left(\theta_{T},x_{T}\right)$ as *structural components* of their respective models. The notion of *similarity* will be defined in the construction of our RECaST framework for transfer learning, presented next.

Denote the forward data-generating representations of $P\left(y_{S}|x_{S}\right)$ and $P\left(y_{T}|x_{T}\right)$, respectively, by

(1) $$Y_{S}=h\left\{f\left(\theta_{S},x_{S}\right),U_{S}\right\}\;\text{and}\;Y_{T}=h\left\{g\left(\theta_{T},x_{T}\right),U_{T}\right\},$$

where ${𝒳}_{S}={𝒳}_{T}=R^{p}$, and $U_{T}$ and $U_{S}$ are independent and identically distributed auxiliary random variables. We give two examples of the h function (for continuous and binary response examples), but the h function is much more general. It is to be understood as any scalar-valued function that relates the covariates to the auxiliary random variable in the fashion of a data generating equation. In fact, in the case of a continuous random variable, the h function can be taken to be the inverse cumulative distribution function, by the probability integral transform. For example, if

$$f\left(\theta_{S},x_{S}\right)=x_{S}^{⊤}\theta_{S},$$

$$h\left(x_{S}^{⊤}\theta_{S},U_{S}\right)=x_{S}^{⊤}\theta_{S}+U_{S},\text{and}$$

$$U_{S}\sim 𝒩(0, 1),$$

then $Y_{S}\sim 𝒩\left(x_{S}^{⊤}\theta_{S},1\right)$. Or in the case of binary outcome data, for example, if

$$f\left(\theta_{S},x_{S}\right)=\text{expit}\left(x_{S}^{⊤}\theta_{S}\right),$$

$$h\left(x_{S}^{⊤}\theta_{S},U_{S}\right)=1\left\{U_{S}<\text{expit}\left(x_{S}^{⊤}\theta_{S}\right)\right\},\;\text{and}$$

$$U_{S}\sim \text{Uniform}(0, 1),$$

then $Y_{S}\sim \text{Bernoulli}\left\{\text{expit}\left(x_{S}^{⊤}\theta_{S}\right)\right\}$, where $\text{expit}(z):=e^{z}/\left(1+e^{z}\right)$. The *similarity* between the source and target that makes this a formulation of a transfer learning problem is determined by how well the structural component $f\left(\theta_{S},x_{T}\right)$ of the source model approximates the structural component $g\left(\theta_{T},x_{T}\right)$ of the target model.

Accordingly, transfer learning should be effective if $\beta :=g\left(\theta_{T},x_{T}\right)/f\left(\theta_{S},x_{T}\right)\approx 1$, and sufficient source data is available for reliable estimation of $\theta_{S}$; in fact, the source and target models are identical if β=1. Assuming $f\left(\theta_{S},x_{T}\right)\neq 0$ *almost surely* (a.s.), it follows a.s. that

(2) $$Y_{T,i}=h\left\{\beta_{i}\cdot f\left(\theta_{S},x_{T,i}\right),U_{T,i}\right\},$$

for $i\in \left\{1,\ldots ,n_{T}\right\}$, where $Y_{T,1},\ldots ,Y_{T,n_{T}}$ is an independent sample of $n_{T}$ target labels with associated features $x_{T,1},\ldots ,x_{T,n_{T}}$, and $\beta_{i}:=g\left(\theta_{T},x_{T,i}\right)/f\left(\theta_{S},x_{T,i}\right)$. The identity given by Equation (2) is further motivated by the fact that, for first-order approximations of the source and target models, if we assume $x_{T,1},\ldots ,x_{T,n_{T}}\overset{\text{iid}}{\sim }{𝒩}_{p}\left(0,I_{p}\right)$, then by Lemma 1 (a well-known result for which we provide a proof in Appendix A, for convenience), $\beta_{i}=\left(x_{T,i}^{⊤}\theta_{T}\right)/\left(x_{T,i}^{⊤}\theta_{S}\right)\sim \text{Cauchy}(\delta ,\gamma )$, with

$$\delta =\frac{\theta_{T}^{⊤}\theta_{S}}{{\left\|\theta_{S}\right\|}^{2}},\;\text{and}$$

$$\gamma =\frac{1}{{\left\|\theta_{S}\right\|}^{2}}\sqrt{{\left\|\theta_{S}\right\|}^{2}{\left\|\theta_{T}\right\|}^{2}-{\left(\theta_{T}^{⊤}\theta_{S}\right)}^{2}}.$$

**Lemma 1** *For any* $a,b\in R^{p}$, *if* $x\sim {𝒩}_{p}\left(0,I_{p}\right)$ *then* $\left(\left.x^{⊤}a\right)/\left(x^{⊤}b\right)\sim \text{Cauchy}(\delta ,\gamma \right)$, *with* $\delta =a^{⊤}b/\|b{\|}^{2}$ *and* $\gamma =\|b{\|}^{-2}\sqrt{\|b{\|}^{2}\|a{\|}^{2}-{\left(a^{⊤}b\right)}^{2}}$.

That being so, while Equation (2) is motivated by a first-order approximation, $f\left(\theta_{S},x_{S}\right)$ and $g\left(\theta_{T},x_{T}\right)$ need *not* share the same structure to implement the RECaST framework described by Equation (2). In fact, Equation (2) does not make any account of $g\left(\theta_{T},x_{T}\right)$; it only assumes that the source model and parameters are available with the target data.

In practice, we assume without loss of generality that features have been centered and scaled to have mean zero and unit variance. Central limit theory supports the Gaussian approximation for more complex, nonlinear models (i.e., for the large p scenarios that characterize modern ML approaches). Specifically, appealing to the Lyapunov or Lindeberg central limit theorem gives Gaussian approximations for the distributions of $x_{T,i}^{⊤}\theta_{S}/{\left\|\theta_{S}\right\|}^{2}$ and $x_{T,i}^{⊤}\theta_{T}/{\left\|\theta_{T}\right\|}^{2}$. For more general assumptions on f and g, first-order approximations motivate $f\left(\theta_{S},x_{T,i}\right)\approx x_{T,i}^{⊤}\theta_{S}$ and $g\left(\theta_{T},x_{T,i}\right)\approx x_{T,i}^{⊤}\theta_{T}$. The edge case with γ→∞ describes a situation in which there is no link between the source and target domains. Assuming γ<∞, the RECaST model specified by Equation (2) with random effect $\beta_{i}\sim \text{Cauchy}(\delta ,\gamma )$ fully characterizes the *similarity* between the source and target domains. In addition to being the exact distribution in the linear model case with Gaussian features, the Cauchy distribution also provides benefit through its heavy tails. This attribute allows $\beta_{i}$ to capture large disparities between source and target data sets, improving the frequentist coverage of resulting prediction sets.

Estimating parameters of Cauchy distributions is a notoriously difficult problem since the heavy tails allow outlying events to happen with relatively high probability (Schuster, 2012). Some estimation procedures focus on estimating solely the location parameter (Zhang, 2010) or the scale parameter (Kravchuk and Pollett, 2012), but rarely both. Fegyverneki (2013) explores the trade-off between using simple robust estimators, for both parameters, which are less asymptotically efficient than the maximum likelihood estimators. Recently, limit theorems are established in Akaoka et al. (2022) for quasi-arithmetic means for point estimation in cases where the strong law of large numbers fails, such as with Cauchy random variables. The fact that the Cauchy distribution appears in our work speaks to the difficulty of a transfer learning problem.

There are three primary advantages of our RECaST transfer learning model formulation in Equation (2) with random effect $\beta_{i}\sim \text{Cauchy}(\delta ,\gamma )$. First, regardless of the complexity of the source model (e.g., $f\left(\theta_{S},\right)$ could represent a DNN with millions of parameters in $\theta_{S}$ trained on extensive source data), RECaST only ever requires estimation of the parameters δ and γ, and perhaps a scale parameter associated with $U_{T,i}$ through $h\left(\cdot ,U_{T,i}\right)$. Existing transfer learning methods require either estimation of $\theta_{T}$ (often via fine-tuning from an estimate of $\theta_{S}$) or learning of $n_{T}+n_{S}$ weights for pooling the source and target data, where $n_{S}$ is the number of source training labels. The scalability of our approach cannot be overstated. Second, RECaST needs no source data, only requiring the estimated source parameters ${\hat{\theta }}_{S}$. Such a feature is vital in applications such as with medical data where privacy constraints place legal and ethical barriers to accessing certain data sets. Third, RECaST naturally facilitates uncertainty quantification of target label predictions via the construction of prediction sets. The following two sections propose a Bayesian framework for estimation of the posterior predictive distribution of target labels in the continuous and binary response settings, respectively.

## 4 Continuous Response Data

### 4.1 Model and Estimation

Assume that $Y_{S,1},\ldots ,Y_{S,n_{S}}$ and $Y_{T,1},\ldots ,Y_{T,n_{T}}$ are mutually independent, continuous random variables generated according to source and target models, respectively, as expressed in Equation (1). Also assume that an estimator $f\left({\hat{\theta }}_{S},x\right)$ is available for any feature vector $x\in {𝒳}_{S}={𝒳}_{T}$, where ${\hat{\theta }}_{S}$ is an estimator of $\theta_{S}$ based on $Y_{S,1},\ldots ,Y_{S,n_{S}}$. In the continuous response setting, a natural choice for the h function in the RECaST model, defined by Equation (2), is the Gaussian innovation formulation,

$$Y_{T,i}=\beta_{i}\cdot f\left({\hat{\theta }}_{S},x_{T,i}\right)+\sigma \cdot U_{T,i},$$

independently for $i\in \left\{1,\ldots ,n_{T}\right\}$, where $U_{T,i}\sim 𝒩(0,1),\sigma >0$ is a scaling parameter to be learned from the target data, and $\beta_{i}\sim \text{Cauchy}(\delta ,\gamma )$.

We specify a canonical prior on (δ,γ,σ) as

$$\pi \left(\delta ,\gamma ,\sigma \right)=𝒩\left(\delta |1,\sigma_{\delta }^{2}\right)\cdot \text{log}\;𝒩\left(\gamma |a,b\right)\cdot \text{log}\;𝒩\left(\sigma |c,d\right).$$

The prior distributions are standard for shape and scale parameters. The hyperparameters for σ can be chosen based on prior information about the target domain. The hyperparameters for δ and γ can be chosen based on prior information about similarity between the source and target data. If the domains are known to be very similar, then the prior on δ may be centered near 1 with a small variance and the prior on γ may be chosen to have a mode near 0 with a small variance. This will result in a prior favoring δ and γ values that encourage $\beta =g\left(\theta_{T},x_{T}\right)/f\left(\theta_{S},x_{T}\right)$ values of 1, which indicates a similar source and target. In practice, to demonstrate the robustness of the RECaST framework and to cover a broad range of transfer learning settings, we choose hyperparameter values that induce diffuse priors. See Appendix C for more details.

A posterior distribution of the parameters (δ,γ,σ) can be expressed as

(3) $$\pi \left(\delta ,\gamma ,\sigma |y_{T,1},\ldots ,y_{T,n_{T}},{\hat{\theta }}_{S}\right)\;=\int_{R}\ldots \int_{R}\pi \left(\delta ,\gamma ,\sigma ,\beta_{1},\ldots ,\beta_{n_{T}}|y_{T,1},\ldots ,y_{T,n_{T}},{\hat{\theta }}_{S}\right)\;d\beta_{1}\ldots d\beta_{n_{T}}\propto \pi (\delta ,\gamma ,\sigma )\cdot \int_{R}\ldots \int_{R}\prod_{i=1}^{n_{T}}\left[𝒩\left\{y_{T,i}|\beta_{i}f\left({\hat{\theta }}_{S},x_{T,i}\right),\sigma^{2}\right\}\cdot \text{Cauchy}\left(\beta_{i}|\delta ,\gamma \right)\right]\;d\beta_{1}\ldots d\beta_{n_{T}}\;=\pi (\delta ,\gamma ,\sigma )\cdot \prod_{i=1}^{n_{T}}\int_{R}𝒩\left\{y_{T,i}|\beta_{i}f\left({\hat{\theta }}_{S},x_{T,i}\right),\sigma^{2}\right\}\cdot \text{Cauchy}\left(\beta_{i}|\delta ,\gamma \right)\;d\beta_{i}\;=\pi (\delta ,\gamma ,\sigma )\cdot \prod_{i=1}^{n_{T}}\int_{R}𝒩\left\{\beta_{i}f\left({\hat{\theta }}_{S},x_{T,i}\right)|y_{T,i},\sigma^{2}\right\}\cdot \text{Cauchy}\left(\beta_{i}|\delta ,\gamma \right)\;d\beta_{i}\;=\pi \left(\delta ,\gamma ,\sigma \right)\cdot \prod_{i=1}^{n_{T}}\int_{R}𝒩\left\{\beta_{i}\left|\frac{y_{T,i}}{f\left({\hat{\theta }}_{S},x_{T,i}\right)}\right.,\frac{\sigma^{2}}{f^{2}\left({\hat{\theta }}_{S},x_{T,i}\right)}\right\}\cdot \frac{\text{Cauchy}\left(\beta_{i}|\delta ,\gamma \right)}{\left|f\left({\hat{\theta }}_{S},x_{T,i}\right)\right|}d\beta_{i},$$

where the univariate integrals in the last expression can be evaluated numerically. Next, the posterior predictive distribution of the label ${\tilde{Y}}_{T}$ associated with some new target feature vector ${\tilde{x}}_{T}$ can be derived as the marginal distribution of

(4) $$\pi \left({\tilde{y}}_{T},\tilde{\beta },\sigma ,\delta ,\gamma |y_{T,1},\ldots ,y_{T,n_{T}},{\hat{\theta }}_{S}\right)\;=𝒩\left\{{\tilde{y}}_{T}|\tilde{\beta }f\left({\hat{\theta }}_{S},{\tilde{x}}_{T}\right),\sigma^{2}\right\}\cdot \pi \left(\tilde{\beta },\sigma ,\delta ,\gamma |y_{T,1},\ldots ,y_{T,n_{T}},{\hat{\theta }}_{S}\right)\;=𝒩\left\{{\tilde{y}}_{T}|\tilde{\beta }f\left({\hat{\theta }}_{S},{\tilde{x}}_{T}\right),\sigma^{2}\right\}\cdot \text{Cauchy}\left(\tilde{\beta }|\delta ,\gamma \right)\cdot \pi \left(\delta ,\gamma ,\sigma |y_{T,1},\ldots ,y_{T,n_{T}},{\hat{\theta }}_{S}\right).$$

### 4.2 Remarks on Implementation

To estimate the posterior distribution given in Equation (3), we implement a random walk Metropolis-Hastings algorithm, numerically solving the univariate integrals with the Julia package QuadGK (Johnson, 2013). Furthermore, by expressing these integrals as expectations with respect to a Gaussian distribution (i.e., the final expression in Equation (3)), we show that they are numerically equivalent to definite integrals from −39 to 39. See Appendix B for the mathematical details of this bound. This substantially reduces the computational overhead for the numerical integration.

We detail our implementation of the Metropolis-Hastings algorithm in Appendix C. The chosen number of iterations and length of the burn-in period can be adjusted based on computational resources. Because Metropolis-Hastings evaluates the likelihood for all target data points for each iteration, the computational complexity is $𝒪\left(n_{T}\cdot n_{\text{iterations}}\right)$, with $n_{\text{iterations}}$ the number of Metropolis-Hastings iterations. The fact that $n_{T}$ is assumed to be small for transfer learning problems mitigates concerns about scalability. Posterior predictive credible sets can be constructed as usual in Bayesian inference, from the highest posterior density regions calculated via the empirical quantiles of the sampled posterior predictive values.

In Algorithm 1, we propose a procedure for drawing samples from the posterior predictive distribution described by Equation (4). Again take ${\tilde{x}}_{T}$ to be the feature vector for a new target data point with label ${\tilde{Y}}_{T}$. With the learned posterior distribution of (δ,γ,σ), we are able to sample from the posterior predictive distribution of ${\tilde{Y}}_{T}$. We first sample $n_{\text{post}}(\delta ,\gamma ,\sigma )$ triplets from the posterior distribution. For *each* of these triplets, we sample $n_{\beta }$ β’s from a Cauchy distribution with location and scale parameters corresponding to the δ and γ sampled from the posterior. Finally, for *each* sampled β we sample $n_{Y}$ ${\tilde{y}}_{T}$’s from the normal distribution with mean and variance determined by ${\tilde{x}}_{T}$, the sampled β, and the sampled σ. This gives a total of $n_{\text{post}}\cdot n_{\beta }\cdot n_{Y}$ samples from the posterior predictive distribution for each new target observation. These samples are used to construct the posterior predictive credible sets as described in Algorithm 1 with a computational complexity of $𝒪\left(n_{\text{post}}\cdot n_{\beta }\cdot n_{Y}\right)$. We discuss our choices for these parameters in Appendix C. We showcase the effectiveness of these proposed computational strategies in a variety of simulation scenarios in Section 6.2.

**Algorithm 1:** RECaST posterior predictive sampling: continuous response data

**Input:** ${\tilde{x}}_{T}$, samples from $\pi \left(\delta ,\gamma ,\sigma |y_{T,1},\ldots ,y_{T,n_{T}},{\hat{\theta }}_{S}\right)$, and sample sizes $n_{\text{post}}$, $n_{\beta }$, and $n_{Y}$
**Output:** A sample of values from $\pi \left({\tilde{y}}_{T}|y_{T,1},\ldots ,y_{T,n_{T}},{\hat{\theta }}_{S}\right)$
**for** i←1 to $n_{\text{post}}$ **do**
$\delta ,\gamma ,\sigma \leftarrow \text{random}\left\{\pi \left(\delta ,\gamma ,\sigma |y_{T,1},\ldots ,y_{T,n_{T}},{\hat{\theta }}_{S}\right)\right\}$
**for** j←1 to $n_{\beta }$ **do**
$\tilde{\beta }\leftarrow \text{random}\left\{\text{Cauchy}\left(\delta ,\gamma \right)\right\}$
**for** k←1 to $n_{Y}$ **do**
${\tilde{Y}}_{T}\leftarrow \text{random}\left[𝒩\left\{\tilde{\beta }f\left({\hat{\theta }}_{S},{\tilde{x}}_{T}\right),\sigma^{2}\right\}\right]$
**end for**
**end for**
**end for**

### 4.3 Theoretical Guarantees

In this section, we establish the asymptotic validity of our proposed posterior predictive credible sets in the case of linear source and target models with independent Gaussian innovations. Here, asymptotic validity means that the empirical coverage of a 1-α level prediction credible set attains 1-α level coverage, asymptotically in $n_{T}$, as described by the result of Theorem 3. Our mathematical proof of this result and of all supporting results are organized in Appendix A.

Suppose that $Y_{S,j}$ follows a Gaussian distribution centered at $x_{S,j}^{⊤}\theta_{S}$, independently for $j\in \left\{1,\ldots ,n_{S}\right\}$. In the class of transfer learning problems we consider, it is assumed that consistent or meaningful estimators are available for all source model parameters, and that ample data/resources are available for estimating them. Accordingly, assume that $n_{S}$ is sufficiently large such that $\theta_{S}$ is regarded as known. Next, assume that $Y_{T,1},\ldots ,Y_{T,n_{T}}\overset{\text{iid}}{\sim }𝒩\left({\tilde{x}}^{⊤}\theta_{T},\sigma^{2}\right)$, for some feature vector $\tilde{x}\in {𝒳}_{T}={𝒳}_{S}$, and $\theta_{T}$ unknown. Leveraging the RECaST transfer learning framework, the likelihood function of (δ,γ) can be expressed as

(5) $$L\left(\delta ,\gamma |y_{T,1},\ldots ,y_{T,n_{T}},\beta_{1},\ldots ,\beta_{n_{T}}\right)=\prod_{i=1}^{n_{T}}\left[𝒩\left\{y_{T,i}|\beta_{i}{\tilde{x}}^{⊤}\theta_{S},\sigma^{2}\right\}\cdot \text{Cauchy}\left(\beta_{i}|\delta ,\gamma \right)\right].$$

We investigate the asymptotic coverage of prediction sets constructed from the RECaST posterior predictive distribution with plugin maximum likelihood estimators (MLEs) $\hat{\delta }$ and $\hat{\gamma }$ for δ and γ, respectively:

$$\pi \left({\tilde{y}}_{T},\tilde{\beta }|y_{1},\ldots ,y_{n_{T}}\right)=𝒩\left({\tilde{y}}_{T}|\tilde{\beta }{\tilde{x}}^{⊤}\theta_{S},\sigma^{2}\right)\cdot \text{Cauchy}(\tilde{\beta }|\hat{\delta },|\hat{\gamma }|).$$

This is the same as considering maximum a posteriori (MAP) estimators for δ and γ with a flat prior π(δ,γ)∝1, and the choice of prior is not so meaningful in the $n_{T}\to \infty$ setting. Recall that in the RECaST framework the $\beta_{1},\ldots ,\beta_{n_{T}}$ that appear in the likelihood function in Equation (5) are iid Cauchy(δ,γ) random effects. Nonetheless, we demonstrate with Lemma 2 that the MLEs $\hat{\delta }$ and $\hat{\gamma }$ converge in probability to fixed points such that

$$\pi \left({\tilde{Y}}_{T},\tilde{\beta }|y_{1},\ldots ,y_{n_{T}}\right)\approx 𝒩\left({\tilde{Y}}_{T}|\tilde{\beta }{\tilde{x}}^{⊤}\theta_{S},\sigma^{2}\right)\cdot 1\left\{\tilde{\beta }=\frac{{\tilde{x}}^{⊤}\theta_{T}}{{\tilde{x}}^{⊤}\theta_{S}}\right\}=𝒩\left({\tilde{Y}}_{T}|{\tilde{x}}^{⊤}\theta_{T},\sigma^{2}\right),$$

as desired. This fact leads to our main theoretical result, Theorem 3, which establishes the asymptotic validity of 1-α level RECaST prediction sets of the form $\left[a_{n_{T}}^{\alpha },b_{n_{T}}^{\alpha }\right]$, with

$$a_{n_{T}}^{\alpha }≔\Phi^{-1}(\alpha /2)\cdot \sigma +\tilde{\beta }\cdot {\tilde{x}}^{⊤}\theta_{S}\;\text{and}$$

$$b_{n_{T}}^{\alpha }≔\Phi^{-1}(1-\alpha /2)\cdot \sigma +\tilde{\beta }\cdot {\tilde{x}}^{⊤}\theta_{S}$$

for any α∈(0,1) and $\tilde{\beta }\sim \text{Cauchy}(\hat{\delta },|\hat{\gamma }|)$.

**Lemma 2** *Assuming* $Y_{T,1},\ldots ,Y_{T,n_{T}}\overset{iid}{\sim }𝒩\left({\tilde{x}}^{⊤}\theta_{T},\sigma^{2}\right)$ *and* $\beta_{1},\ldots ,\beta_{n_{T}}\overset{iid}{\sim }\text{Cauchy}(\delta ,\gamma )$, *independently*, *the MLEs of* δ *and* γ *for* Equation (5) *satisfy*

$$\hat{\delta }⟶\frac{{\tilde{x}}^{⊤}\theta_{T}}{{\tilde{x}}^{⊤}\theta_{S}}\;\text{and}\;\hat{\gamma }⟶0$$

*in probability as* $n_{T}\to \infty$.

**Theorem 3** *Assume that* ${\tilde{Y}}_{T}\sim 𝒩\left({\tilde{x}}^{⊤}\theta_{T},\sigma^{2}\right)$. *Then*, *for any* α∈(0, 1),

$$P\left({\tilde{Y}}_{T}\in \left[a_{n_{T}}^{\alpha },b_{n_{T}}^{\alpha }\right]\right)=\int_{a_{n_{T}}^{\alpha }}^{b_{n_{T}}^{\alpha }}\frac{1}{\sigma \sqrt{2\pi }}e^{-\frac{1}{2\sigma^{2}}{\left({\tilde{y}}_{T}-{\tilde{x}}^{⊤}\theta_{T}\right)}^{2}}d{\tilde{y}}_{T}⟶1-\alpha$$

*in probability as* $n_{T}\to \infty$.

In Section 6, we provide empirical evidence that RECaST achieves near nominal coverage even in more practical, small $n_{T}$ settings, trained on target data that arise from both linear and non-linear models. In the empirical investigations in Section 6, we relax the assumptions of known σ and the availability of repeated samples from a fixed feature vector $\tilde{x}$.

## 5 Binary Response Data

### 5.1 Model and Estimation

Assume that $Y_{S,1},\ldots ,Y_{S,n_{S}}$ and $Y_{T,1},\ldots ,Y_{T,n_{T}}$ are mutually independent, Bernoulli random variables generated according to source and target models, respectively, as expressed in Equation (1). Also assume that an estimator $f\left({\hat{\theta }}_{S},x\right)$ is available for any feature vector $x\in {𝒳}_{S}={𝒳}_{T}$, where ${\hat{\theta }}_{S}$ is an estimator of $\theta_{S}$ based on $Y_{S,1},\ldots ,Y_{S,n_{S}}$. In the binary response setting, a natural choice for the h function in the RECaST model, defined by Equation (2), is the logistic model formulation,

$$Y_{T,i}=1\left[U_{T,i}<\text{expit}\left\{\beta_{i}\cdot f\left({\hat{\theta }}_{S},x_{T,i}\right)\right\}\right],$$

with $U_{T,i}\sim \text{Uniform}(0,1)$ independently for $i\in \left\{1,\ldots ,n_{T}\right\}$ and $\beta_{i}\sim \text{Cauchy}(\delta ,\gamma )$.

As in the continuous setting, the RECaST posterior distribution of the parameters can be constructed as

$$\pi \left(\delta ,\gamma |y_{T,1},\ldots ,y_{T,n_{T}},{\hat{\theta }}_{S}\right)\;=\int_{R}\ldots \int_{R}\pi \left(\delta ,\gamma ,\beta_{1},\ldots ,\beta_{n_{T}}|y_{T,1},\ldots ,y_{T,n_{T}},{\hat{\theta }}_{S}\right)\;d\beta_{1}\ldots d\beta_{n_{T}}\propto \pi \left(\delta ,\gamma \right)\cdot \prod_{i=1}^{n_{T}}\int_{R}\text{Bernoulli}\left[y_{T,i}|\text{expit}\left\{\beta_{i}f\left({\hat{\theta }}_{S},x_{T,i}\right)\right\}\right]\cdot \text{Cauchy}\left(\beta_{i}|\delta ,\gamma \right)\;d\beta_{i},$$

and the posterior predictive distribution of the label ${\tilde{Y}}_{T}$ associated with some new target feature vector ${\tilde{x}}_{T}$ can be derived as the marginal distribution of

(6) $$\pi \left({\tilde{y}}_{T},\tilde{\beta },\delta ,\gamma |y_{T,1},\ldots ,y_{T,n_{T}},{\hat{\theta }}_{S}\right)\;=\text{Bernoulli}\left[{\tilde{y}}_{T}|\text{expit}\left\{\tilde{\beta }f\left({\hat{\theta }}_{S},{\tilde{x}}_{T}\right)\right\}\right]\cdot \text{Cauchy}\left(\tilde{\beta }|\delta ,\gamma \right)\cdot \pi \left(\delta ,\gamma |y_{T,1},\ldots ,y_{T,n_{T}},{\hat{\theta }}_{S}\right).$$

We specify a canonical prior on (δ,γ) as

$$\pi \left(\delta ,\gamma \right)=𝒩\left(\delta |1,\sigma_{\delta }^{2}\right)\cdot \text{log}\;𝒩\left(\gamma |a,b\right),$$

with diffuse choices of the hyperparameters $\sigma_{\delta },a,b$. A similar description to that in Section 4.1 of the choice of priors holds here.

A 1-α level RECaST prediction credible set, denoted $\Gamma_{n_{T}}^{\alpha }$, for binary response values is constructed as

(7) $$\Gamma_{n_{T}}^{\alpha }=\left\{\begin{array}{ll} \{0\}, & \text{if}\;\tilde{p}<1-\tilde{p}\;\text{and}\;1-\alpha \leq 1-\tilde{p}\\ \{1\}, & \text{if}\;1-\tilde{p}\leq \tilde{p}\;\text{and}\;1-\alpha \leq \tilde{p}\\ \{0, 1\}, & \text{else}, \end{array}\right.$$

where $\tilde{p}≔\pi \left({\tilde{y}}_{T}=1|y_{T,1},\ldots ,y_{T,n_{T}},{\hat{\theta }}_{S}\right)$.

### 5.2 Remarks on Implementation

The RECaST transfer learning computations in the binary response setting follow analogously to those described in Section 4.2. For completeness, Algorithm 2 specifies the procedure we propose for drawing samples from the posterior predictive distribution described by Equation (6).

**Algorithm 2:** RECaST posterior predictive sampling: binary response data

**Input: ${\tilde{x}}_{T}$**, samples from $\pi \left(\delta ,\gamma |y_{T,1},\ldots ,y_{T,n_{T}},{\hat{\theta }}_{S}\right)$, and sample sizes $n_{\text{post}}$, $n_{\beta }$, and $n_{Y}$
**Output:** A sample of values from $\pi \left(\tilde{y}|y_{T,1},\ldots ,y_{T,n_{T}},{\hat{\theta }}_{S}\right)$
**for** i←1 to $n_{\text{post}}$ **do**
$\delta ,\gamma \leftarrow \text{random}\left\{\pi \left(\delta ,\gamma |y_{T,1},\ldots ,y_{T,n_{T}},{\hat{\theta }}_{S}\right)\right\}$
**for** j←1 to $n_{\beta }$ **do**
$\tilde{\beta }\leftarrow \text{random}\left\{\text{Cauchy}\left(\delta ,\gamma \right)\right\}$
**for** k←1 to $n_{Y}$ **do**
${\tilde{Y}}_{T}\leftarrow \text{random}\left(\text{Bernoulli}\left[\text{expit}\left\{\tilde{\beta }f\left({\hat{\theta }}_{S},{\tilde{x}}_{T}\right)\right\}\right]\right)$
**end for**
**end for**
**end for**

## 6 Simulation Study

### 6.1 Objectives and Setup

In this section, we examine the finite sample performance of RECaST through simulations on synthetic data. We consider continuous and binary responses with source models corresponding to linear (RECaST LM) and logistic (RECaST GLM) regression, respectively, as well as a DNN (RECaST DNN) source model for both response types. We assess the empirical coverage with respect to the nominal coverage level of the prediction sets. If the method is calibrated, the empirical coverage will match the nominal significance level. We use the terms *empirical coverage* and *observed coverage* interchangeably.

We generate the synthetic data from linear and logistic regressions with source parameter vector $\theta_{S}$ and target parameter vector $\theta_{T}$, with p=50 features (including an intercept). The features are generated from the standard Gaussian distribution, $x_{S,i},x_{T,j}\sim {𝒩}_{p-1}\left(0,I_{p-1}\right)$. We fix the source data generating parameters $\theta_{S}$. The source data generating parameters are set to $\theta_{S}=(-a,b)$ where $a,b\in R^{25}$ have components independently sampled from Uniform(0.75, 5) and then fixed for all simulations. The *similarity* of source and target domains is controlled by choosing the value of $\sigma_{\text{TL}}>0$ in constructing $\theta_{T}=\theta_{S}+\epsilon$ with $\epsilon \sim {𝒩}_{p}\left(0,\sigma_{\text{TL}}^{2}I_{p}\right)$. We consider values of $\sigma_{\text{TL}}^{2}\in \{0, 0.25, 1,4\}$. Setting $\sigma_{\text{TL}}^{2}=0$ corresponds to $\theta_{T}=\theta_{S}$, i.e., no difference between the source and target distributions. Since the source parameters lie within [−5, −0.75] ∪ [0.75, 5], a variance of $\sigma_{\text{TL}}^{2}=4$ allows for significant differences between $\theta_{T}$ and $\theta_{S}$. We fix the source sample size at $n_{S}=1000$, and vary the target sample size $n_{T}$ to examine performance when $p<n_{T}\left(n_{T}=100, 250\right),p$ is near $n_{T}\left(n_{T}=40, 60\right)$, and $p>n_{T}\left(n_{T}=20\right)$. We simulate 300 source and target data sets for each of these 20 combinations of $\sigma_{\text{TL}}^{2}$ and $n_{T}$ values, and implement the estimation procedures described in Sections 4.2 and 5.2. See Appendix C for additional details about the specifics of our implementations.

We compare to a linear model baseline (LM) which is built only on the target data. Another baseline for comparison is constructed from training a DNN on the target data, without any transfer learning, and we compare RECaST to other state-of-the-art transfer learning approaches. We build a DNN on the source data and fine-tune the last layer on the target data (Unfreeze DNN); this is often referred to as *freezing* the weights of the source DNN and *unfreezing* the last layer. See Appendix D for details on this procedure. Other state-of-the-art transfer learning approaches that we compare RECaST to include TransRF (Gu et al., 2022), a source-free method that adapts a random forest model built in the source domain to target data, and glmtrans (Tian and Feng, 2022), which is based on penalized GLMs and designed to mitigate the impact of negative transfer. Unlike RECaST and TransRF, glmtrans requires the source data to be available during the training of the model. In the continuous setting, we compare to the source-free methods outlined by Tripuraneni et al. (2021). We compare to both their first order method (MTL FO) and their method of moments approach (MTL MoM). Note that while this method does not require the source data when learning the target model, it does require that the source parameters were learned following their formulation whereas RECaST is agnostic to the choice of source model. In the binary setting, we compare RECaST to the regularized logistic regression (Wiens) approach of Wiens et al. (2014). This approach uses the combined source and target EHR data to build a regularized model for disease prediction – similar to the real data application we consider in Section 7, but with the disadvantage that Wiens requires access to the source data (while RECaST does not). In the binary setting, we also compare RECaST to the adversarial transfer learning approach WDGRL (Shen et al., 2018), which also requires access to the source data.

Throughout this section, all DNN training proceeds by setting aside a portion of the training data to be used as a calibration data set. The final DNN parameters are chosen from the epoch with the minimum calibration loss to improve generalizability to out-of-sample test sets. Additional details/specifications for our DNN training procedures are provided in Appendix D.

### 6.2 Continuous Response Results

**Figure 1:**
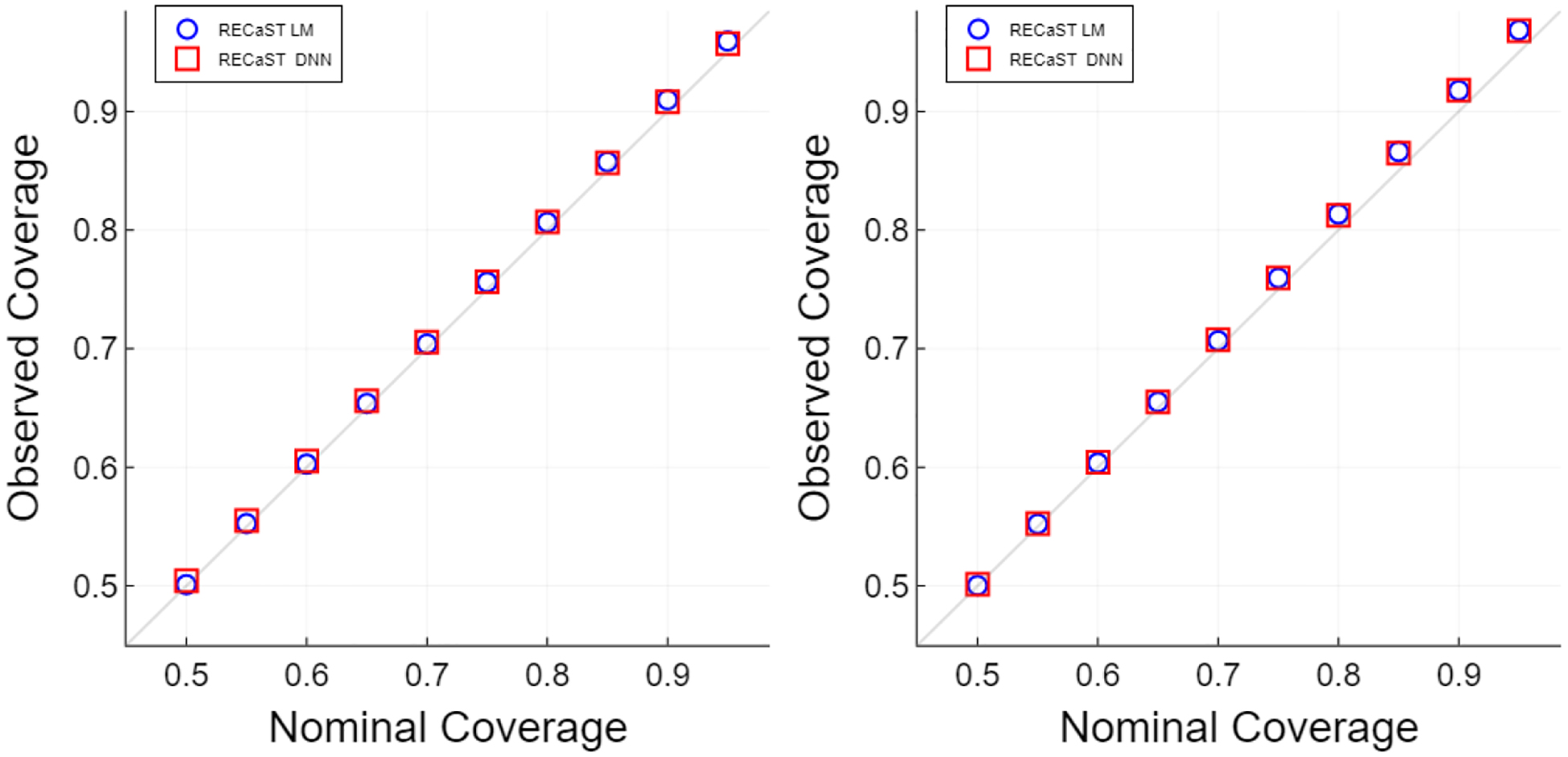
Reliability curves of the nominal coverage versus the empirical coverage, averaged over 300 source and target data sets for each setting; the out-of-sample test sets each contain 250 observations. The left panel shows an easy setting: $n_{T}=100$ and $\sigma_{\text{TL}}^{2}=0.25$. The right panel shows a difficult setting: $n_{T}=20$ and $\sigma_{\text{TL}}^{2}=4$.

Table 1 and Figure 1 summarize the performance of the prediction uncertainty quantification provided by our RECaST framework implementations. Table 1 presents the empirical coverage for 95% nominal level prediction sets for each simulation setting. Recall that the empirical coverage should ideally match the nominal significance for a given level; an empirical coverage greater than the nominal coverage level corresponds to a conservative interval estimate. RECaST methods consistently provide empirical coverage at or slightly above nominal levels, supporting the use of RECaST for inference on out-of-sample target domain predictions. Additionally, Figure 1 plots empirical versus nominal coverage for the $\sigma_{\text{TL}}^{2}=0.25,n_{T}=100$ and $\sigma_{\text{TL}}^{2}=4,n_{T}=20$ settings at a grid of nominal levels. The empirical coverages consistently achieve the associated nominal levels or are slightly conservative.

Out-of-sample root mean squared errors (RMSEs) for all methods, averaged over 300 source and target data sets are presented in Table 2. The LM provides the best prediction when the sample size is large since in this case it correctly specifies the data generating model and has enough data to estimate the parameters. There is a large decrease in performance, noted by the increase in RMSE, when $n_{T}<p$ and a generalized inverse has to be used for parameter estimation. As expected, the performance of DNN deteriorates as the target sample size decreases. Note that the baseline DNN is overparameterized, which leads to it having higher RMSEs than the baseline LM.

Interestingly, the RECaST RMSE values remain consistent for each value of $\sigma_{\text{TL}}^{2}$, regardless of sample size, suggesting that RECaST is appropriate even when the target sample size is so small as to preclude a target-only analysis. Meanwhile, Unfreeze DNN exhibits an increase in RMSE for each value of $\sigma_{\text{TL}}^{2}$ as $n_{T}$ decreases. As source and target become more dissimilar, both Unfreeze DNN and RECaST exhibit similar increases in RMSE. In fact, with $n_{T}=250$ and $\sigma_{\text{TL}}^{2}=4$, the target-only DNN outperforms both RECaST methods. This setting is the most prone to negative transfer: the target sample size is large enough to learn meaningful DNN parameters, and the source *and* target data distributions differ greatly, making transfer difficult. We see this phenomenon with the target only LM as well; with a sample size of $n_{T}=40$, both RECaST methods outperform the LM except for when the source and target are most dissimilar. When $n_{T}=20$, the RECaST methods outperform the LM in all settings. This highlights a situation where transfer learning is necessary because the target domain lacks sufficient data to efficiently estimate the target parameters, even with a correctly specified model.

The MTL FO and MTL MoM both see increases in RMSE as the source and target become more dissimilar and see a larger increase in RMSE as the target sample size decreases. Interestingly, in this simulation these two methods have the same performance when there are more target sample points than there are features. While in some settings with larger target sample sizes the Unfreeze DNN slightly outperforms RECaST, it has larger standard errors and fails to provide uncertainty quantification. We find that TransRF sometimes performs well but with high RMSE variance. We were not able to evaluate TransRF when the target sample size was 20 as the software gave NA values instead of predictions without an accompanying error message. While glmtrans sometimes has smaller RMSE than RECaST, recall that it requires access to the source data and that only RECaST provides uncertainty quantification for predictions.

### 6.3 Binary Response Results

Table 3 shows that RECaST procedures, again, provide near nominal coverages with low standard errors across sample sizes in the binary response setting.

Compared to the other approaches, RECaST provides substantial inferential advantages that are robust to small target sample sizes and large dissimilarity between source and target. Recall from Equation (7) that prediction sets in the binary response setting are determined entirely by the Bernoulli probability of observing label 1. Thus, we can construct prediction sets for the DNN, Unfreeze DNN, and Wiens methods, as well. When a method fails to discriminate between the two labels at level 1-α (e.g., when the Bernoulli probability of success and failure are *both below* 1-α), then the prediction set must include both labels to attain the 1-α level. In such cases, as observed for the Wiens method in various settings in Table 3, the prediction set achieves 100% empirical coverage, but is unhelpful for prediction.

Table 4 provides the area under the receiver operator characteristic curve (AUC) for all methods and simulation settings. In all settings except one, RECaST DNN outperforms all other methods. We see similar patterns here as in the continuous setting. The RECaST models consistently report the highest AUC, with low standard errors across sample sizes. In contrast, the AUC of DNN and Unfreeze DNN drastically declines as $n_{T}$ decreases. As expected, the AUC of all transfer learning methods decreases as the difficulty of the problem increases with larger values of $\sigma_{\text{TL}}^{2}$. RECaST DNN and WDGRL frequently outperform other methods; however, WDGRL requires access to the source data, an important limitation that is unrealistic in many applications. WDGRL crashed with a sample size of $n_{T}=20$, so we are unable to evaluate its performance in these settings.

The benefits to coverage properties and predictive performance of the RECaST method are especially important in the binary response case. This demonstrates that RECaST can be used even when the linearity assumption of Lemma 1 is violated.

### 6.4 Robustness of RECaST

#### 6.4.1 Over-Parameterized RECaST DNN

In all previous simulations, the true data generating mechanisms are linear or logistic models. To test the robustness of RECaST, we now consider a more complex case where data are generated from neural networks. We generate data from a neural network with a densely connected input layer of size $\ell_{1}=(p,10)$ and then pass through a ReLU activation function to an output layer of size $\ell_{2}=(10, 1)$, where there are p=50 features generated as described in Section 6.1. For the binary response data, we append a sigmoid activation function to the end of the output layer. While the source and target data generating networks share architectures, we consider two relationships between the source and target neural network parameters.

In our first set of simulations, as in Section 6.1, we take the parameters of the source neural network to be $\theta_{S}\overset{\text{iid}}{\sim }U(-1, 1)$ and define the parameters of the target neural network as $\theta_{T}=\theta_{S}+\epsilon$ with $\epsilon \sim {𝒩}_{p\times 10+10\times 1}(0,0.025I)$. For a continuous outcome, Table 5 shows that the RECaST DNN methods have the lowest RMSE for all sample sizes. Both RECaST methods outperform the target-only DNN across all settings, even when the target sample size is large $\left(n_{T}=250\right)$. The glmtrans method performs similarly to RECaST LM but worse than RECaST DNN. For all sample sizes, the RECaST framework produces wide posterior predictive intervals with 100% observed coverage for the 95% nominal confidence level – see Table 12 in Appendix E.. This greater than nominal coverage demonstrates RECaST will be conservative but reliable. Indeed, the observed over-coverage is safer than narrower intervals centered around incorrect values with below nominal coverage. For a binary outcome, Table 6 reveals that both RECaST methods outperform the target-only DNN for all sample sizes. This shows robustness to negative transfer. The performance of the RECaST methods is stable across target sample sizes in this setting, with stable AUCs and standard errors, whereas other methods degrade in performance as the target sample size decreases. Table 7 shows the empirical coverages of each method at the 75% nominal level. Only the RECaST GLM, RECaST DNN, and Wiens methods provide conservative coverage values for all sample sizes whereas the other methods tend to under-cover the true labels as the target sample size decreases.

#### 6.4.2 Orthogonal Source and Target Data Generating Model Parameters

In our second set of simulations, we set the source and target weight matrices to be orthogonal, i.e., $\theta_{S}^{⊤}\theta_{T}=0$. For a continuous outcome, Table 8 shows that RECaST again provides consistent predictive performance across target sample sizes. For small sample sizes, both RECaST methods outperform the target-only LM and DNN. The unfreeze DNN and glmtrans also perform well, but we mention again that they do not provide uncertainty quantification of predictions. Table 13 in Appendix E shows that RECaST provides conservative coverage intervals which, again, is a safe feature in this difficult transfer learning setting. For a binary outcome, Table 9 shows that RECaST again outperforms the target-only DNN in realistic settings where the target sample size is small. The RECaST methods have consistent AUCs across target sample sizes whereas other methods deteriorate as the sample size decreases. Table 10 shows that only RECaST GLM, RECaST DNN, and the Wiens method provide conservative uncertainty quantification for all target sample sizes at the 75% nominal level.

Overall, the results presented in this section show that RECaST is robust to negative transfer under more complex data generating mechanisms. In all cases, the RECaST methods outperformed the target-only DNN while boasting conservative predictive coverage intervals when the target sample size is small. In Appendix F we explore other relationships between the source and target data when the data generating mechanism is a (generalized) linear model. These include orthogonality of source and target parameters and the target data having more features than the source.

## 7. eICU Data

The eICU Collaborative Research Database (Pollard et al., 2018) is a publicly available database of ICU encounters across multiple hospitals in the United States, making it well-suited for imitating transfer learning settings using real data. In the spirit of the transfer learning application in Wiens et al. (2014), we focus on correctly diagnosing physiological shock for newly admitted ICU patients. We define a binary response variable as the indicator of the event that a patient experienced shock upon ICU admission, using a combination of Internal Classification of Diseases 10 (ICD-10) codes: R57 Shock, not elsewhere classified; R58 Hemorrhage, not elsewhere classified; or R65.21 Severe sepsis with septic shock. Features are limited to baseline variables measured at admission. While the simulations of Section 6 explicitly link the source and target data through the data generation process, the similarity between source and targets defined in our eICU data application is unknown.

We consider 19 features including patient demographics, Acute Physiology Score III variables, and Glasgow Coma Scale test. Descriptions of these features can be found in Table 22 in Appendix H. The data consist of measurements on 45,945 patients across 156 unique hospitals. Only 700 of these patients were diagnosed with shock upon admission. No individual hospital had enough positive cases to be reliably used as a source data set. To curate a balanced data set, we take all 700 patients with shock and randomly sample an additional 700 patients with no shock. Next, 80% of the hospitals associated with our sampled 1,400 patients are randomly selected to define the ‘source hospital’. The source data set consists of all ICU encounters at the ‘source hospital’. Of the remaining 20% of hospitals, half are randomly assigned to the ‘target training hospital’, and the other half define a ‘target testing hospital’. Notice that this procedure splits hospitals rather than patients; the source data set may not consist of 80% of patients. The target training and target testing data sets typically contain 80 to 130 patients each.

**Figure 2:**
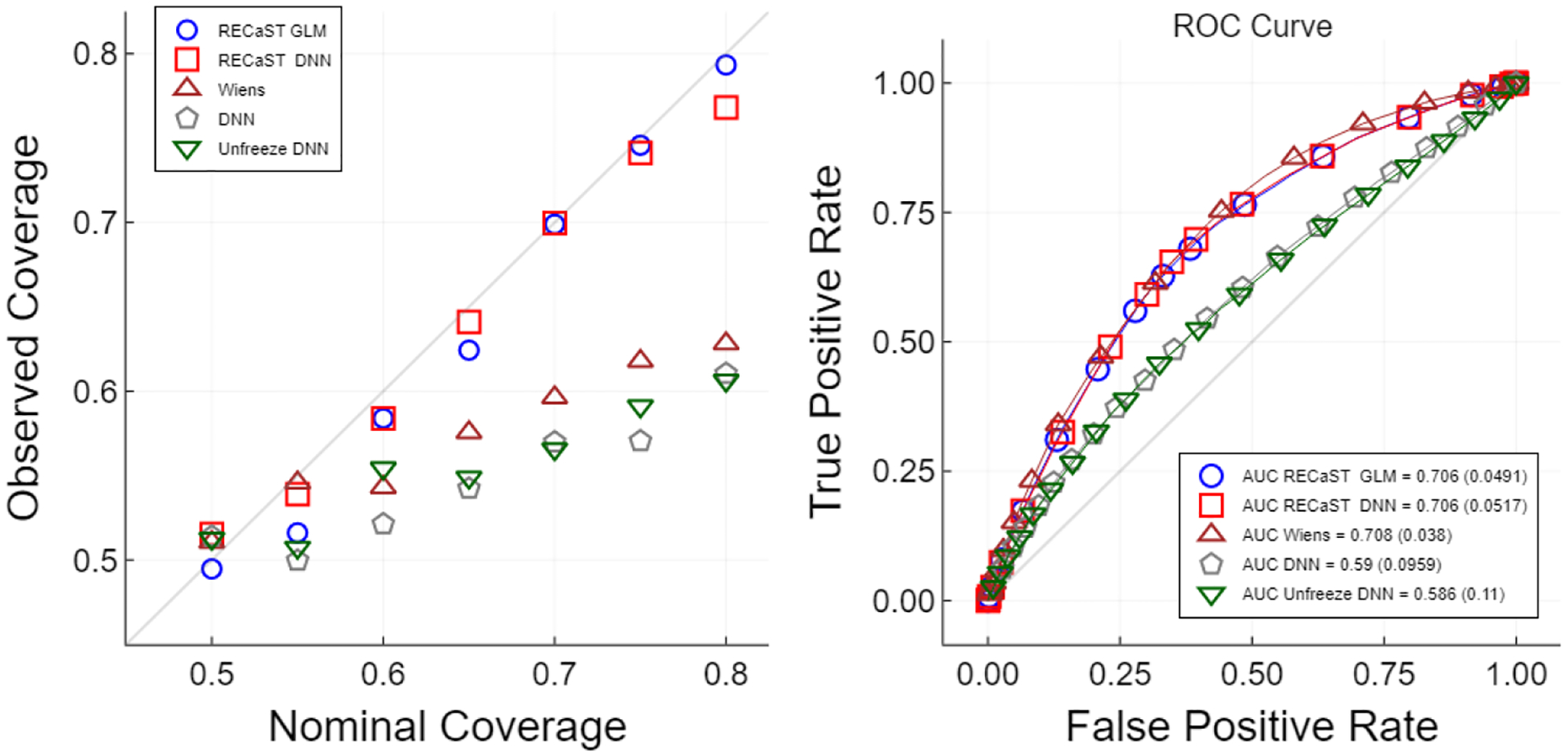
The left panel displays the reliability curve of the nominal versus empirical out-of-sample coverage of prediction sets averaged over 300 target-testing data sets; the right panel reports the out-of-sample receiver operating characteristic (ROC) curve averaged pointwise over 300 target-testing data sets. The legend also reports the AUC (standard error) averaged over the same 300 target-testing data sets. Note that we cut the reliability curve at a nominal coverage of 0.8 because there are very few observations with higher coverage, undermining the reliability of coverage estimation at higher nominal levels.

We repeat the described sampling procedure 300 times, to imitate 300 transfer learning scenarios from real data. A logistic regression model and a DNN model are trained on each of the 300 source data sets, and all previously considered binary response transfer learning methods are implemented on the target data sets. To boost the performance of the source DNN model, the architecture of the DNN is chosen from a set of candidate architectures by maximizing AUC, averaged over 100 of the source data sets; additional details are provided in Appendix D. In Figure 2, we report the empirical coverage and AUC.

Because the real data generating model is unknown we consider two additional target-only models to test for negative transfer. We compare to a GLM and a Gaussian process (GP) trained only on the target data. In this setting, they perform worse than all of the transfer learning methods, with the GLM achieving an AUC of 0.606 and the GP achieving an AUC of 0.512. Plots for the TransRF, glmtrans, and WDGRL methods can be found in Appendix G. The AUCs of glmtrans and WDGRL were 0.68 and 0.708, respectively. RECaST has similar predictive performance to Wiens and WDGRL but without requiring access to the source data, and it outperforms the DNN and Unfreeze DNN approaches. Pairing RECaST with either the logistic regression or DNN source models produced near optimal average AUC, with respect to the average AUC values of 0.704 and 0.708, respectively, for the source logistic regression model and source DNN model. Figure 2 also demonstrates that RECaST generally produces prediction sets that achieve their nominal level of coverage for target test response values, even for non-linear models with non-Gaussian data, whereas the other approaches do not.

In addition to splitting the data into source and target by hospital, we explore making this division based on other features. First, we take the target data to be all patients aged 51 and under. This split resulted in approximately 20% of the patients in the target data and 80% in the source data. Second, we take the target data to be all patients aged 55 and under. This age was chosen because 20% of the patients *that experienced shock* are aged 55 and under. Third, we take the target data to be all female patients, which account for about 45% of the data. This more even split between source and target will be a good test for negative transfer. Finally, we take the target data to be all patients who are not Caucasian, corresponding to roughly 20% of the data.

Table 11 shows the average AUC, AUC standard error, and average empirical coverage at the 80% nominal level summarized over 300 target training and testing data sets. While the standard errors are large, we see that the average AUC of RECaST is larger than that of the target-only methods in all but one setting. The only instance in which RECaST has smaller AUC is when the target data consist of the female patients. This may be due to the similar sample sizes between the source and target for this particular setting, as we demonstrated in the synthetic data simulations that RECaST is most advantageous when the target sample size is small. The RECaST AUCs are within a standard error of Wiens, glmtrans, and WDGRL, but RECaST does not require access to the source data. We see that the empirical coverages for the RECaST method are near the 80% nominal value; the Wiens and glmtrans methods are more conservative when the data are split by age. The TransRF method reports coverage lower than the 80% nominal level in all settings. This analysis demonstrates a general use case for RECaST as a clinical tool across a broad range of scenarios.

## 8. Concluding Remarks

The RECaST framework is adaptable to virtually any source model that makes predictions, and can accommodate both continuous and binary responses. The source data themselves are not required, which is a significant advantage when legal or ethical barriers to access of source data sets exist, e.g., due to privacy concerns. Unlike other transfer learning methods, RECaST always provides uncertainty quantification through prediction sets. Our conclusions are supported by both theoretical justifications and performance in simulation studies on synthetic and real data using linear and two-layer neural network source models.

The RECaST framework may be extended in several directions to accommodate the complexity of EHR data. Broadening RECaST to handle differing feature spaces between source and target hospitals would allow for it to be applied in more general settings. As EHR databases are updated, it would be useful to perform online transfer learning. Patient clinical notes are also frequently available in EHR data and have been used by other transfer learning approaches (e.g., Si and Roberts, 2020). However, transfer learning approaches that combine quantitative and text features to create a unified patient representation are currently lacking. Another promising direction is to study RECaST framework formulations for multi-class classification. One such formulation would be to specify the h function in Equation (2) as

$$h\left\{f\left(\theta_{S},x_{S}\right),U_{S}\right\}=\sum_{k=1}^{K}k\cdot 1\left[U_{S}\in \Delta_{k}\left\{f\left(\theta_{S},x_{S}\right)\right\}\right],$$

where K is the number of classes and $U_{S}\sim \text{Uniform}(\Delta )$ with $\Delta_{1},\ldots ,\Delta_{K}$ – all functions of $f\left(\theta_{S},x_{S}\right)$ – being triangular regions that form a partition of the simplex Δ over the multi-class outcome space (e.g., see, Jacob et al., 2021; Williams, 2021).

## Figures and Tables

**Table 1:** Empirical coverage (standard error) at the 95% nominal level, averaged over 300 source and target data sets for each setting; the out-of-sample test sets each contain 250 observations. All reported values are multiplied by 100.

$n_{T}$	$\sigma_{\text{TL}}^{2}$	RECaST LM	RECaST DNN
250	0	96(1.8)	94(1.9)
	0.25	95(1.9)	95(1.9)
	1	95(1.9)	95(1.8)
	4	95(2.0)	95(1.9)
100	0	96(1.8)	94(2.1)
	0.25	96(1.8)	96(2.0)
	1	96(1.8)	96(1.8)
	4	96(1.8)	96(1.9)
60	0	97(2.2)	94(2.6)
	0.25	96(1.9)	96(1.8)
	1	96(1.8)	96(1.8)
	4	96(1.8)	96(1.8)
40	0	97(2.1)	94(3.3)
	0.25	96(2.4)	96(2.4)
	1	96(2.6)	96(2.5)
	4	96(2.8)	96(2.8)
20	0	98(1.8)	95(3.0)
	0.25	97(2.6)	97(2.8)
	1	97(2.6)	97(2.8)
	4	97(2.7)	97(2.9)

**Table 2:** Out-of-sample RMSE (standard error) averaged over 300 source and target data sets for each setting; the out-of-sample test sets each contain 250 observations.

$n_{T}$	$\sigma_{T}^{2}$	LM	DNN	RECaST LM	RECaST DNN	Unfreeze DNN	TransRF	glmtrans	MTL FO	MTL MoM
250	0	0.57(0.03)	2.8(0.38)	0.52(0.027)	1.2(0.090)	0.58(0.038)	14(1.5)	0.56(0.026)	1.9(0.07)	1.9(0.07)
	0.25	0.57(0.03)	2.9(0.37)	3.6(0.43)	3.8(0.4)	2.8(0.42)	14(1.4)	0.56(0.027)	1.9(0.5)	1.9(0.5)
	1	0.57(0.03)	3.1(0.43)	7.1(0.86)	7.2(0.84)	5.5(0.90)	14(1.5)	0.56(0.027)	2.0(1.0)	2.0(1.0)
	4	0.57(0.03)	3.7(0.52)	14(1.7)	14(1.7)	11(1.8)	17(2.6)	0.56(0.027)	2.5(1.7)	2.5(1.7)
100	0	0.71(0.07)	8.9(1.6)	0.52(0.022)	1.2(0.095)	0.81(0.095)	22(12)	0.69(0.047)	2.4(0.2)	2.4(0.2)
	0.25	0.71(0.06)	9.1(1.3)	3.6(0.42)	3.8(0.40)	3.2(0.57)	28(75)	0.73(0.068)	2.4(0.7)	2.4(0.7)
	1	0.71(0.06)	9.4(1.3)	7.1(0.85)	7.2(0.83)	6.3(1.1)	23(16)	0.74(0.075)	2.5(1.2)	2.5(1.2)
	4	0.71(0.06)	11(1.52)	14(1.7)	14(1.7)	13(2.1)	31(34)	0.74(0.073)	3.1(2.1)	3.1(2.1)
60	0	1.3(0.27)	14(2.5)	0.52(0.025)	1.2(0.11)	1.5(0.29)	46(73)	0.75(0.05)	4.0(0.87)	4.0(0.88)
	0.25	1.3(0.23)	13(1.6)	3.6(0.43)	3.8(0.42)	3.7(0.78)	48(99)	1.2(0.21)	4.0(1.2)	4.0(1.2)
	1	1.3(0.23)	14(1.8)	7.1(0.87)	7.2(0.86)	6.8(1.3)	54(85)	1.7(0.38)	4.2(2.1)	4.2(2.1)
	4	1.3(0.23)	16(2.6)	14(1.8)	14(1.8)	13(2.0)	51(59)	3.0(0.85)	5.1(3.5)	5.1(3.5)
40	0	10(2.1)	17(2.6)	0.52(0.024)	1.2(0.089)	1.8(0.61)	74(64)	0.78(0.063)	11(2.1)	10(1.9)
	0.25	11(1.8)	17(2.4)	3.6(0.41)	3.8(0.40)	4.1(1.1)	62(74)	2.5(0.51)	11(2.1)	10(1.9)
	1	11(2.0)	18(2.5)	7.2(0.83)	7.3(0.83)	7.6(2.2)	69(110)	4.7(1.1)	11(2.4)	11(2.2)
	4	12(2.0)	20(2.9)	14(1.7)	14(1.7)	14(3.0)	150(540)	8.9(2.3)	13(3.0)	13(2.9)
20	0	18(1.8)	21(1.8)	0.54(0.03)	1.2(0.11)	2.5(2.2)	-	0.81(0.078)	18(2.0)	17(1.6)
	0.25	18(1.8)	21(1.8)	3.7(0.44)	3.9(0.42)	4.7(2.5)	-	3.4(0.39)	18(1.9)	18(1.7)
	1	18(1.9)	22(2.0)	7.3(0.90)	7.4(0.90)	8.5(3.5)	-	6.7(0.8)	19(2.1)	18(1.9)
	4	21(2.4)	24(2.7)	15(1.8)	15(1.8)	16(4.0)	-	6.7(0.8)	21(2.5)	20(2.4)

**Table 3:** Empirical coverage (standard error) at the 95% nominal level, averaged over 300 source and target data sets for each setting; the out-of-sample test sets each contain 250 observations. All reported values are multiplied by 100.

$n_{T}$	$\sigma_{\text{TL}}^{2}$	DNN	RECaST GLM	RECaST DNN	Wiens	Unfreeze DNN	TransRF	glmtrans	WDGRL
250	0	84(9.5)	95(0.78)	96(0.090)	100(0)	91(8.4)	89(12)	98(4.4)	95(4.4)
	0.25	89(7.5)	95(0.82)	96(0.13)	100(0)	93(6.8)	88(11)	98(2.6)	94(4.8)
	1	91(6.2)	95(0.65)	96(0.14)	100(0)	95(4.5)	87(9.5)	98(3.6)	86(6.0)
	4	93(6.4)	95(0.40)	95(0.39)	99(1.5)	95(4.8)	86(11)	97(4.1)	75(7.6)
100	0	80(12)	96(1.1)	96(0.25)	100.0(0)	90(8.7)	68(22)	96(6.4)	96(3.4)
	0.25	83(11)	95(1.3)	96(0.27)	100(0)	92(7.8)	78(3.0)	94(6.2)	93(4.2)
	1	88(8.2)	95(1.2)	96(0.35)	100(4.6)	94(5.9)	69(18)	94(8.5)	89(5.7)
	4	92(6.2)	95(0.81)	95(0.90)	95(13)	94(10.0)	49(20)	83(4.6)	89(2.3)
60	0	80(13)	95(1.2)	96(0.54)	100(0.0)	92(6.4)	65(19)	93(9.3)	95(3.7)
	0.25	77(17)	95(1.3)	96(0.67)	100(0.0)	91(8.1)	64(22)	88(12)	95(3.1)
	1	80(21)	95(1.1)	95(0.867)	100(0.49)	94(6.1)	63(19)	88(15)	90(4.7)
	4	84(15)	95(0.59)	95(1.0)	96.8(4.7)	93(8.7)	58(19)	89(11)	80(6.9)
40	0	68(23)	95(1.6)	96(0.86)	100(0.0)	89(11)	60(20)	88(14)	95(5.5)
	0.25	72(20)	95(1.6)	96(0.99)	100(0.0)	90(7.9)	55(25)	81(16)	95(4.1)
	1	76(19)	94(1.5)	95(1.2)	100(0.53)	89(8.7)	59(19)	85(14)	89(5.9)
	4	77(25)	94(1.4)	94(1.1)	97(3.2)	90(7.2)	63(20)	78(14)	78(7.4)
20	0	67(22)	95(1.1)	96(0.78)	100(0.0)	85(17)	-	86(15)	-
	0.25	75(16)	95(1.1)	95(0.98)	100(0.0)	86(14)	-	68(15)	-
	1	75(16)	95(0.84)	95(1.1)	100(0.55)	86(17)	-	63(18)	-
	4	72(13)	95(0.47)	94(0.99)	98(1.5)	80(18)	-	66(17)	-

**Table 4:** Out-of-sample AUC (standard error) averaged over 300 source and target data sets for each setting; the out-of-sample test sets each contain 250 observations. All reported values are multiplied by 100.

$n_{T}$	$\sigma_{\text{TL}}^{2}$	DNN	RECaST GLM	RECaST DNN	Wiens	Unfreeze DNN	TransRF	glmtrans	WDGRL
250	0	95(1.7)	98(2.1)	98(0.61)	80(3.5)	97(1.2)	66(13)	97(2.0)	97(0.95)
	0.25	95(1.6)	97(2.3)	98(0.89)	80(3.9)	97(1.2)	69(10)	96(1.6)	97(1.3)
	1	94(1.5)	93(3.8)	96(1.5)	79(3.9)	95(1.7)	66(11)	97(1.8)	95(1.5)
	4	95(1.7)	84(5.5)	89(2.8)	76(4.0)	89(3.0)	67(12)	97(1.6)	88(3.2)
100	0	85(7.9)	96(2.2)	98(0.64)	81(4.2)	96(2.2)	47(19)	87(9.3)	98(0.66)
	0.25	83(9.6)	95(2.7)	97(1.0)	80(4.1)	95(1.9)	18(14)	81(5.3)	97(1.0)
	1	84(8.4)	92(3.8)	95(1.4)	79(4.4)	93(2.4)	48(20)	81(5.5)	95(1.4)
	4	82(11)	83(4.7)	89(3.1)	74(4.3)	87(4.8)	49(20)	83(4.6)	89(2.3)
60	0	72(13)	96(1.9)	98(1.0)	80(4.3)	94(5.2)	49(20)	83(4.6)	89(2.3)
	0.25	74(11)	94(2.5)	97(1.4)	80(4.3)	94(2.34)	36(21)	74(5.1)	97(0.78)
	1	75(10)	90(3.5)	95(1.7)	78(4.1)	91(6.0)	33(20)	74(5.3)	95(1.9)
	4	72(11)	83(4.0)	89(3.3)	73(4.7)	84(8.0)	29(18)	75(5.6)	89(2.4)
40	0	68(11)	96(1.6)	98(1.1)	80(3.8)	94(4.5)	27(16)	83(16)	97(1.1)
	0.25	68(11)	94(2.2)	97(1.3)	80(4.0)	92(6.7)	19(15)	67(4.9)	97(1.2)
	1	65(12)	90(3.0)	95(1.9)	78(3.9)	89(7.9)	32(18)	69(5.7)	95(1.7)
	4	67(12)	82(4.1)	89(3.5)	74(4.2)	80(12)	31(18)	69(4.9)	89(3.2)
20	0	60(8.7)	96(1.7)	97(1.4)	80(3.9)	89(10)	-	81(18)	-
	0.25	61(9.1)	94(2.1)	97(1.9)	79(4.2)	87(13)	-	62(5.1)	-
	1	60(9.5)	90(2.7)	94(2.5)	77(4.5)	83(14)	-	60(5.0)	-
	4	62(8.2)	82(3.5)	88(3.0)	72(5.0)	77(11)	-	63(5.0)	-

**Table 5:** Out of sample RMSE (standard error) averaged over 300 source and target data sets when the generating models are neural networks and the target model parameters are generated as $\theta_{T}=\theta_{S}+\epsilon$ with ϵ~𝒩(0,0.025I). The out-of-sample test sets each contain 250 observations. All reported values are multiplied by 100.

$n_{T}$	LM	DNN	RECaST LM	RECaST DNN	Unfreeze DNN	TransRF	glmtrans	MTL FO	MTL MoM
250	2.9(0.18)	3.1(0.22)	2.9(0.16)	2.1(0.15)	3(0.22)	3.9(0.53)	2.7(0.15)	3.1(0.21)	3.1(0.2)
100	3.8(0.36)	4.2(0.51)	2.9(0.16)	2.1(0.15)	3.3(0.37)	5.5(2.6)	2.7(0.18)	3.8(0.37)	3.9(0.38)
60	6.7(1.6)	5.1(0.56)	2.9(0.16)	2.1(0.15)	3.7(0.65)	12(10)	2.8(0.19)	6.3(1.3)	6.4(1.4)
40	6.1(1.1)	5.4(0.56)	2.9(0.16)	2.1(0.15)	4(0.67)	170(560)	2.8(0.18)	7.4(1.4)	6.8(1.5)
20	4.8(0.38)	5.8(0.44)	3(0.21)	2.2(0.17)	4.6(0.93)	-	2.9(0.26)	7.2(0.83)	4.9(0.42)

**Table 6:** Out-of-sample AUC (standard error) averaged over 300 source and target data sets when the generating models are neural networks and the target model parameters are generated as $\theta_{T}=\theta_{S}+\epsilon$ with ϵ~𝒩(0,0.025I). The out-of-sample test sets each contain 250 observations. All reported values are multiplied by 100.

$n_{T}$	DNN	RECaST GLM	RECaST DNN	Wiens	Unfreeze DNN	TransRF	glmtrans	WDGRL
250	85(3.3)	92(1.6)	91(1.9)	75(4)	89(2.8)	64(13)	86(2.5)	89(1.7)
100	73(9.5)	92(1.6)	91(1.8)	75(3.5)	85(5.9)	46(18)	76(4.4)	89(1.7)
60	66(9.7)	92(1.7)	91(2)	75(4.4)	81(9)	29(22)	71(6.9)	89(2.2)
40	62(9)	92(1.8)	91(1.8)	76.0(3.5)	78(11)	24(16)	67(8)	89(1.9)
20	58(7.9)	92(1.8)	91(2)	76.0(3.6)	72(14)	-	56(6.4)	-

**Table 7:** Empirical coverage (standard error) at the 75% nominal level for a binary response, averaged over 300 source and target data sets when the generating models are neural networks and the target model parameters are generated as $\theta_{T}=\theta_{S}+\epsilon$ with ϵ~𝒩(0,0.025I). The out-of-sample test sets each contain 250 observations. All reported values are multiplied by 100.

$n_{T}$	DNN	RECaST GLM	RECaST DNN	Wiens	Unfreeze DNN	TransRF	glmtrans	WDGRL
250	68(13)	98(2)	97(4.1)	88(6.6)	71(13)	72(12)	80(11)	70(7.9)
100	63(12)	95(6.1)	94(6.7)	86(7.5)	70(13)	58(15)	72(12)	64(16)
60	59(12)	90(14)	91(10)	88(7.6)	66(14)	53(26)	75(16)	69(12)
40	58(12)	87(15)	87(13)	88(5.2)	63(15)	61(13)	69(17)	67(15)
20	55(12)	81(17)	81(18)	89(6.6)	59(14)	-	57(17)	-

**Table 8:** Out of sample RMSE (standard error) averaged over 300 source and target data sets when the source and target neural network weight matrices are orthogonal. The out-of-sample test sets each contain 250 observations. All reported values are multiplied by 100.

$n_{T}$	LM	DNN	RECaST LM	RECaST DNN	Unfreeze DNN	TransRF	glmtrans	MTL FO	MTL MoM
250	0.93(0.43)	1.1(0.19)	2.9(0.91)	3(0.98)	1.6(0.45)	3.5(1.1)	1(0.41)	1.3(0.69)	1.3(0.69)
100	1.2(0.56)	2.3(0.51)	2.8(0.89)	3(0.97)	1.8(0.52)	5.9(3.2)	1.3(0.52)	1.6(0.86)	1.6(0.86)
60	2.2(1.1)	3.2(0.88)	2.8(0.89)	3(0.96)	2.1(0.62)	7.7(6.3)	1.9(0.7)	2.6(1.4)	2.7(1.3)
40	2.8(0.94)	3.8(0.97)	2.8(0.89)	3(0.97)	2.4(0.8)	21(24)	2.3(0.61)	4.5(1.3)	3.4(1.4)
20	3.7(1.1)	4.5(1.1)	2.9(0.91)	3.1(1)	2.9(0.89)	-	2.6(0.83)	6.6(1.1)	3.8(1.1)

**Table 9:** Out-of-sample AUC (standard error) averaged over 300 source and target data sets when the source and target neural network weight matrices are orthogonal. The out-of-sample test sets each contain 250 observations. All reported values are multiplied by 100.

$n_{T}$	DNN	RECaST GLM	RECaST DNN	Wiens	Unfreeze DNN	TransRF	glmtrans	WDGRL
250	94(1.8)	76(17)	87(11)	72(6.7)	90(6.3)	69(9.5)	95(2.1)	89(11)
100	84(7.2)	77(17)	87(11)	67(7.4)	87(7.9)	43(16)	79(5.6)	87(11)
60	74(10)	78(16.0)	87(11)	64(9)	83(11)	33(19)	66(11)	83(14)
40	68(10)	79(15)	87(11)	64(9.7)	81(13)	24(17)	61(8.4)	87(12)
20	60(9.6)	83(12)	87(12)	65(11)	77(14)	-	55(5.7)	-

**Table 10:** Empirical coverage (standard error) at the 75% nominal level for a binary response, averaged over 300 source and target data sets when the source and target neural network weight matrices are orthogonal. The out-of-sample test sets each contain 250 observations. All reported values are multiplied by 100.

$n_{T}$	DNN	RECaST GLM	RECaST DNN	Wiens	Unfreeze DNN	TransRF	glmtrans	WDGRL
250	68(18)	100(0)	98.0(1.8)	77(29)	70(14)	73(9.5)	79(9.7)	65(19)
100	65(14)	100(0)	99.0(2.2)	80(25)	69(15)	58(13)	72(10)	64(17)
60	63(12)	88(3.5)	94.0(6.2)	80(23)	67(15)	57(16)	68(16)	53(17)
40	60(13)	89(9.9)	89(15)	75(20)	63(15)	61(20)	63(18)	67(15)
20	56(12)	81(15)	81(17)	78(22)	61(15)	-	56(15)	-

**Table 11:** Out-of-sample AUC (standard error) [empirical coverage at the 80% nominal level] averaged over 300 target training and testing data sets for each target data setting of the eICU data. All reported values are multiplied by 100.

	Age ≤ 51	Age ≤ 55	Female	Non-Caucasian
Target only GLM	71(6.9) [0.74]	71(6.0) [0.73]	70(4.2) [0.74]	67(6.4) [0.72]
Target only GP	66(16) [0.78]	67(13) [0.73]	64(11) [0.67]	61(11) [0.74]
Target only DNN	69(8.1) [0.72]	69(7.0) [0.69]	68(5.2) [0.70]	67(8.4) [0.69]
RECaST GLM	73(6.8) [0.78]	72(6.2) [0.78]	69(4.5) [0.77]	71(6.0) [0.84]
RECaST DNN	73(6.8) [0.82]	72(6.1) [0.78]	69(4.5) [0.79]	71(6.1) [0.83]
Unfreeze DNN	69(9.0) [0.75]	69(7.8) [0.73]	68(5.2) [0.72]	66(8.4) [0.70]
Wiens	73(6.5) [0.85]	73(5.7) [0.87]	70(4.5) [0.79]	71(6.6) [0.87]
glmtrans	71(7.1) [0.85]	71(5.5) [0.84]	70(4.7) [0.76]	66(7.2) [0.82]
TransRF	54(14) [0.69]	59(13) [0.71]	66(7.6) [0.72]	56(12) [0.69]
WDGRL	72(7.2) [0.76]	71(6.7) [0.73]	70(3.8) [0.74]	73(6.4) [0.80]

**Figure 3:**
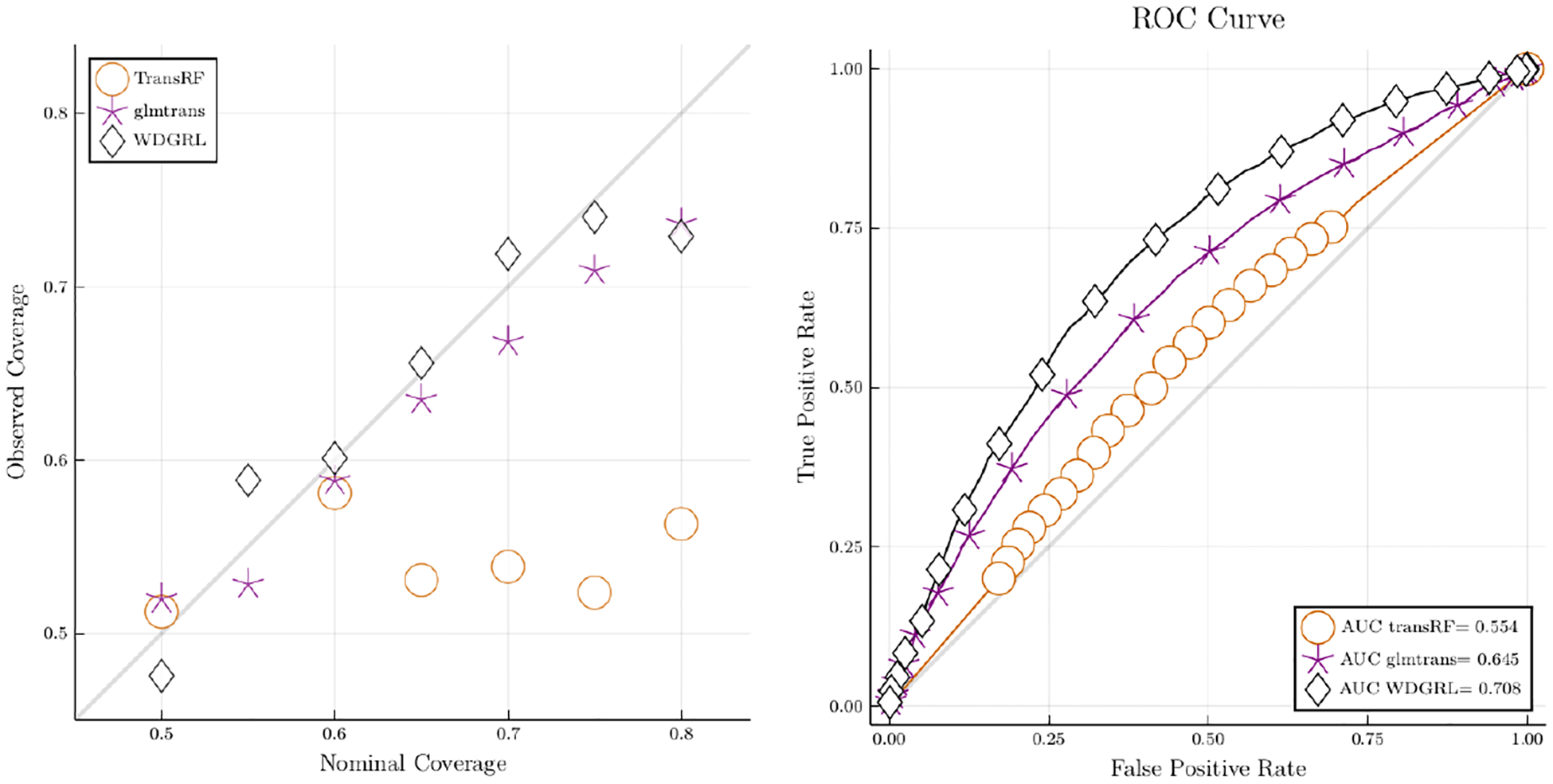
Results for TransRF, glmtrans, and WDGRL on the eICU data set. The left panel displays the reliability curve of the nominal versus empirical out-of-sample coverage of prediction sets averaged over 300 target-testing data sets; the right panel reports the out-of-sample receiver operating characteristic (ROC) curve averaged pointwise over 300 target-testing data sets. The legend also reports the AUC (standard error) averaged over the same 300 target-testing data sets. Note that we cut the reliability curve at a nominal coverage of 0.8 because there are very few observations with higher coverage, undermining the reliability of coverage estimation at higher nominal levels.

## Appendix A. Proofs

**Proof** [Proof of Lemma 1] It is well-established (see, e.g., Hinkley, 1969) that if $V\sim 𝒩\left(0,\sigma_{V}^{2}\right)$ and $W\sim 𝒩\left(0,\sigma_{W}^{2}\right)$ with correlation coefficient ρ, then

(8)
$$\frac{V}{W}\sim \text{Cauchy}\left(\frac{\rho \sigma_{V}}{\sigma_{W}},\frac{\sigma_{V}}{\sigma_{W}}\sqrt{1-\rho^{2}}\right).$$

Accordingly, since

$$\left[\begin{array}{l} a^{⊤}\\ b^{⊤} \end{array}\right]x\sim 𝒩\left(\left[\begin{array}{l} 0\\ 0 \end{array}\right],\left[\begin{array}{ll} a^{⊤}a & a^{⊤}b\\ b^{⊤}a & b^{⊤}b \end{array}\right]\right),$$

it follows that $x^{⊤}a\sim 𝒩\left(0,a^{⊤}a\right),x^{⊤}b\sim 𝒩\left(0,b^{⊤}b\right)$, and $\rho =\left(a^{⊤}b\right)/(\|b\|\|a\|)$. The result follows from Equation (8) by taking $V=x^{⊤}a$ and $W=x^{⊤}b$. ■

Before proceeding directly to the proof of Lemma 2, the following necessary supporting result is stated and proved.

**Lemma 4** *The MLEs* of γ *and* δ *for* Equation (5)*, respectively, are*

$$\hat{\gamma }=\frac{\sum_{i=1}^{n_{T}}\left(v_{i}-\overset{‾}{v}\right)\left(y_{i}-{\overset{‾}{y}}_{T}\right)}{{\tilde{x}}^{⊤}\theta_{S}\sum_{i=1}^{n_{T}}{\left(v_{i}-\overset{‾}{v}\right)}^{2}}\;\text{and}$$

$$\hat{\delta }=\frac{{\overset{‾}{y}}_{T}}{{\tilde{x}}^{⊤}\theta_{S}}-\overset{‾}{v}\cdot \hat{\gamma },$$

*where* $v_{i}=\left(\beta_{i}-\delta \right)/\gamma$ *for* $i\in \left\{1,\ldots ,n_{T}\right\},\overset{‾}{v}≔\sum_{i=1}^{n_{T}}v_{i}/n_{T}$ *and* ${\overset{‾}{y}}_{T}≔\sum_{i=1}^{n_{T}}y_{T,i}/n_{T}$.

**Proof** [Proof of Lemma 4] After the change of variables $v_{i}=\left(\beta_{i}-\delta \right)/\gamma$ for $i\in \left\{1,\ldots ,n_{T}\right\}$, the likelihood function in Equation (5) takes the form

$$\prod_{i=1}^{n_{T}}\left[𝒩\left\{y_{T,i}|\left(\gamma v_{i}+\delta \right){\tilde{x}}^{⊤}\theta_{S},\sigma^{2}\right\}\cdot \text{Cauchy}\left(v_{i}|0, 1\right)\right].$$

Taking partial derivatives with respect to δ and γ gives the first-order conditions

$$\sum_{i=1}^{n_{T}}\left\{\frac{y_{T,i}}{{\tilde{x}}^{⊤}\theta_{S}}-\gamma v_{i}-\delta \right\}=0$$

$$\sum_{i=1}^{n_{T}}\left\{\frac{y_{T,i}}{{\tilde{x}}^{⊤}\theta_{S}}-\gamma v_{i}-\delta \right\}v_{i}=0.$$

Solving this system yields the MLEs in Lemma 4. ■

**Proof** [Proof of Lemma 2] With the assumptions that $Y_{T,1},\ldots ,Y_{T,n_{T}}\overset{\text{iid}}{\sim }𝒩\left({\tilde{x}}^{⊤}\theta_{T},\sigma^{2}\right)$ independent of $V_{1},\ldots ,V_{n_{T}}\overset{\text{iid}}{\sim }\text{Cauchy}(0,1)$, first, define the following notations:

$$Y≔\left(\begin{array}{c} Y_{T,1}\\ \vdots \\ Y_{T,n_{T}} \end{array}\right),\;\bar{Y}≔{\overset{‾}{Y}}_{T}\cdot 1_{n_{T}},\;{\overset{‾}{Y}}_{T}≔\frac{1}{n_{T}}\sum_{i=1}^{n_{T}}Y_{T,i},$$

and

$$V≔\left(\begin{array}{c} V_{1}\\ \vdots \\ V_{n_{T}} \end{array}\right),\;\bar{V}≔\overset{‾}{V}\cdot 1_{n_{T}},\;\overset{‾}{V}≔\frac{1}{n_{T}}\sum_{i=1}^{n_{T}}V_{i},$$

where $1_{n_{T}}$ is an $n_{T}$-dimensional column vector with every component having value 1.

By the Cauchy-Schwarz inequality,

$$\left|\hat{\gamma }\right|=\frac{\left|\sum_{i=1}^{n_{T}}\left(V_{i}-\overset{‾}{V}\right)\left(Y_{i}-{\overset{‾}{Y}}_{T}\right)\right|}{\left|{\tilde{x}}^{⊤}\theta_{S}\right|\sum_{i=1}^{n_{T}}{\left(V_{i}-\overset{‾}{V}\right)}^{2}}\leq \frac{\left\|Y-\bar{Y}{\|}_{2}\right\|V-\bar{V}{\|}_{2}}{\left|{\tilde{x}}^{⊤}\theta_{S}\right|\|V-\bar{V}{\|}_{2}^{2}}=\frac{\|Y-\bar{Y}{\|}_{2}}{\left|{\tilde{x}}^{⊤}\theta_{S}\right|\|V-\bar{V}{\|}_{2}},$$

where $\|\cdot {\|}_{2}$ is the Euclidean norm. We first need to establish the fact that square-root sums of independent, centered, and squared Cauchy random variables grow in value at the rate of at least $n_{T}^{\alpha +\frac{1}{2}}$ for any α∈(0, 1/2). Accordingly, for any ε>0 and any α∈(0, 1/2),

(9)
$$P\left(\|V-\bar{V}{\|}_{2}<n_{T}^{\alpha +\frac{1}{2}}\varepsilon^{-1}\right)=P\left(\|V-\bar{V}{\|}_{2}^{2}<n_{T}^{2\alpha +1}\varepsilon^{-2}\right)\;=P\left(\sum_{i=1}^{n_{T}}V_{i}^{2}-n_{T}{\overset{‾}{V}}^{2}<n_{T}^{2\alpha +1}\varepsilon^{-2}\right)\;\leq P\left(\sum_{i=1}^{n_{T}}V_{i}^{2}-n_{T}^{1+\alpha }<n_{T}^{2\alpha +1}\varepsilon^{-2}\right)+P\left(-n_{T}{\overset{‾}{V}}^{2}<-n_{T}^{1+\alpha }\right)\;=P\left(\sum_{i=1}^{n_{T}}V_{i}^{2}<n_{T}^{2\alpha +1}\varepsilon^{-2}+n_{T}^{1+\alpha }\right)+P\left(|\overset{‾}{V}|>n_{T}^{\frac{\alpha }{2}}\right)\;\leq P\left(\sum_{i=1}^{n_{T}}V_{i}^{2}<n_{T}^{2\alpha +1}\left\{\varepsilon^{-2}+1\right\}\right)+2F_{V}\left(-n_{T}^{\alpha /2}\right),$$

where $F_{V}(\cdot )$ is the Cauchy(0, 1) distribution function. The first term vanishes for any α∈(0, 1/2) as $n_{T}\to \infty$ by Lemma 2.1 in Eicker (1985), and the second term vanishes as $n_{T}\to \infty$ by the definition of a distribution function.

Next, in order to show the convergence of both MLEs, we need that $n_{T}^{\alpha /2}\hat{\gamma }\to 0$ in probability as $n_{T}\to \infty$. Our argument goes as follows. For any ε>0 and any α∈(0,1/2),

$$P\left(|\hat{\gamma }|>n_{T}^{-\alpha /2}\varepsilon \right)\leq P\left(\frac{\|Y-\bar{Y}{\|}_{2}}{\left|{\tilde{x}}^{⊤}\theta_{S}\right|\|V-\bar{V}{\|}_{2}}>\frac{n_{T}^{(1+\alpha )/2}}{n_{T}^{(1+\alpha )/2}}\frac{\varepsilon }{n_{T}^{\alpha /2}}\right)\;\leq P\left(\frac{\|Y-\bar{Y}{\|}_{2}}{\left|{\tilde{x}}^{⊤}\theta_{S}\right|}>n_{T}^{(1+\alpha )/2}\right)+P\left(\frac{1}{\|V-\bar{V}{\|}_{2}}>\frac{\varepsilon }{n_{T}^{\alpha +1/2}}\right)\;=P\left(\frac{\|Y-\bar{Y}{\|}_{2}^{2}}{\sigma^{2}}>\frac{{\left|{\tilde{x}}^{⊤}\theta_{S}\right|}^{2}}{\sigma^{2}}n_{T}^{1+\alpha }\right)+P\left(\|V-\bar{V}{\|}_{2}<n_{T}^{\alpha +\frac{1}{2}}\varepsilon^{-1}\right).$$

Denoting $S≔\|Y-\bar{Y}{\|}_{2}^{2}/\sigma^{2}\sim \chi_{n_{T}-1}^{2}$, and applying the Chernoff bound to the first quantity in the last expression gives, for any t<1/2,

$$P\left(S>\frac{{\left|{\tilde{x}}^{⊤}\theta_{S}\right|}^{2}}{\sigma^{2}}n_{T}^{1+\alpha }\right)\leq (1-2t{)}^{-\left(n_{T}-1\right)/2}\text{exp}\;\left\{-\frac{t{\left|{\tilde{x}}^{⊤}\theta_{S}\right|}^{2}}{\sigma^{2}}n_{T}^{1+\alpha }\right\}.$$

Choosing t=1/4 yields the bound

$$P\left(|\hat{\gamma }|>n_{T}^{-\alpha /2}\varepsilon \right)\leq e^{-n_{T}^{1+\alpha }\cdot \frac{1}{2}\left(\frac{1}{2\sigma^{2}}{\left|{\tilde{x}}^{⊤}\theta_{S}\right|}^{2}-n_{T}^{-\alpha }+n_{T}^{-1-\alpha }\right)}+P\left(\|V-\bar{V}{\|}_{2}<n_{T}^{\alpha +\frac{1}{2}}\varepsilon^{-1}\right).$$

Thus, by Equation (9), it follows that $n_{T}^{\alpha /2}\hat{\gamma }\to 0$ in probability as $n_{T}\to \infty$. This fact implies that $\hat{\gamma }\to 0$ in probability as $n_{T}\to \infty$, and is needed to prove the asymptotic convergence of $\hat{\delta }$, next.

Since $Y_{T,1},\ldots ,Y_{T,n_{T}}\overset{\text{iid}}{\sim }𝒩\left({\tilde{x}}^{⊤}\theta_{T},\sigma^{2}\right)$, it follows that ${\overset{‾}{Y}}_{T}={\tilde{x}}^{⊤}\theta_{T}+\sigma n_{T}^{-\frac{1}{2}}U$, where U~𝒩(0, 1). That being so, for any ε>0 and any α∈(0,1/2),

$$P\left(\left|\hat{\delta }-\frac{{\tilde{x}}^{⊤}\theta_{T}}{{\tilde{x}}^{⊤}\theta_{S}}\right|>\varepsilon \right)=P\left\{\left|\frac{1}{{\tilde{x}}^{⊤}\theta_{S}}\left({\tilde{x}}^{⊤}\theta_{T}+\sigma n_{T}^{-\frac{1}{2}}U\right)-\overset{‾}{V}\hat{\gamma }-\frac{{\tilde{x}}^{⊤}\theta_{T}}{{\tilde{x}}^{⊤}\theta_{S}}\right|>\varepsilon \right\}\;=P\left(\left|\frac{\sigma }{{\tilde{x}}^{⊤}\theta_{S}}n_{T}^{-\frac{1}{2}}U-\overset{‾}{V}\hat{\gamma }\right|>\varepsilon \right)\leq P\left(\left|\frac{\sigma }{{\tilde{x}}^{⊤}\theta_{S}}n_{T}^{-\frac{1}{2}}U\right|>\frac{\varepsilon }{2}\right)+P\left(|\overset{‾}{V}\hat{\gamma }|>\frac{\varepsilon }{2}\right)\;=2\Phi \left\{-n_{T}^{\frac{1}{2}}\cdot \varepsilon \left|{\tilde{x}}^{⊤}\theta_{S}\right|/(2\sigma )\right\}+P\left(|\overset{‾}{V}|>n_{T}^{\alpha /2}/2\right)+P\left(|\hat{\gamma }|>n_{T}^{-\alpha /2}\varepsilon \right)\;=2\Phi \left\{-n_{T}^{\frac{1}{2}}\cdot \varepsilon \left|{\tilde{x}}^{⊤}\theta_{S}\right|/(2\sigma )\right\}+2F_{V}\left(-n_{T}^{\alpha /2}/2\right)+P\left(|\hat{\gamma }|>n_{T}^{-\alpha /2}\varepsilon \right),$$

where Φ(·) is the standard Gaussian distribution function. The first two terms in the last expression vanish by the definition of a distribution function, and the third term vanishes by the same because we previously established that $n_{T}^{\alpha /2}\hat{\gamma }\to 0$ in probability as $n_{T}\to \infty$. Hence, $\hat{\delta }\to {\tilde{x}}^{⊤}\theta_{T}/\left({\tilde{x}}^{⊤}\theta_{S}\right)$ in probability as $n_{T}\to \infty$. ■

**Proof** [Proof of Theorem 3] Our argument begins with direct evaluation of the probability that ${\tilde{Y}}_{T}\sim 𝒩\left({\tilde{x}}^{⊤}\theta_{T},\sigma^{2}\right)$ is contained in the interval $\left[a_{n_{T}}^{\alpha },b_{n_{T}}^{\alpha }\right]$, and it finishes by applying the result of Lemma 2.

$$P\left({\tilde{Y}}_{T}\in \left[a_{n_{T}}^{\alpha },b_{n_{T}}^{\alpha }\right]\right)=\int_{a_{n_{T}}^{\alpha }}^{b_{n_{T}}^{\alpha }}\frac{1}{\sigma \sqrt{2\pi }}e^{-\frac{1}{2\sigma^{2}}{\left({\tilde{y}}_{T}-{\tilde{x}}^{⊤}\theta_{T}\right)}^{2}}d{\tilde{y}}_{T}=\;\Phi \left(\frac{b_{n_{T}}^{\alpha }-{\tilde{x}}^{⊤}\theta_{T}}{\sigma }\right)-\Phi \left(\frac{a_{n_{T}}^{\alpha }-{\tilde{x}}^{⊤}\theta_{T}}{\sigma }\right),$$

where Φ(·) is the standard Gaussian distribution function. We will first demonstrate that Φ(W)→1-α/2, with

$$W≔\frac{b_{n_{T}}^{\alpha }-{\tilde{x}}^{⊤}\theta_{T}}{\sigma }\;=\Phi^{-1}\left(1-\frac{\alpha }{2}\right)+\frac{1}{\sigma }\left(\tilde{\beta }\cdot {\tilde{x}}^{⊤}\theta_{S}-{\tilde{x}}^{⊤}\theta_{T}\right)\sim \text{Cauchy}\left\{\Phi^{-1}\left(1-\frac{\alpha }{2}\right)+\frac{1}{\sigma }\left(\hat{\delta }\cdot {\tilde{x}}^{⊤}\theta_{S}-{\tilde{x}}^{⊤}\theta_{T}\right),\left|\frac{\hat{\gamma }}{\sigma }{\tilde{x}}^{⊤}\theta_{S}\right|\right\}$$

since $\tilde{\beta }\sim \text{Cauchy}(\hat{\delta },|\hat{\gamma }|)$.

For any ϵ>0,

$$P(|\Phi (W)-(1-\alpha /2)|<\epsilon )=P(1-\alpha /2-\epsilon <\Phi (W)<1-\alpha /2+\epsilon )\;=P\left\{\Phi^{-1}(1-\alpha /2-\epsilon )<W<\Phi^{-1}(1-\alpha /2+\epsilon )\right\}\;=F_{W}\left\{\Phi^{-1}(1-\alpha /2+\epsilon )\right\}-F_{W}\left\{\Phi^{-1}(1-\alpha /2-\epsilon )\right\},$$

where $F_{W}(\cdot )$ is the Cauchy distribution function associated with W. Then,

$$F_{W}\left\{\Phi^{-1}(1-\alpha /2+\epsilon )\right\}=\frac{1}{2}+\frac{1}{\pi }\text{arctan}\;\left\{\frac{c_{1}-\left(\hat{\delta }\cdot {\tilde{x}}^{⊤}\theta_{S}-{\tilde{x}}^{⊤}\theta_{T}\right)/\sigma }{\left|\hat{\gamma }\cdot {\tilde{x}}^{⊤}\theta_{S}\right|/\sigma }\right\},$$

with $c_{1}≔\Phi^{-1}(1-\alpha /2+\epsilon )-\Phi^{-1}(1-\alpha /2)>0$, and similarly,

$$F_{W}\left\{\Phi^{-1}(1-\alpha /2-\epsilon )\right\}=\frac{1}{2}+\frac{1}{\pi }\text{arctan}\;\left\{\frac{c_{2}-\left(\hat{\delta }\cdot {\tilde{x}}^{⊤}\theta_{S}-{\tilde{x}}^{⊤}\theta_{T}\right)/\sigma }{\left|\hat{\gamma }\cdot {\tilde{x}}^{⊤}\theta_{S}\right|/\sigma }\right\},$$

with $c_{2}≔\Phi^{-1}(1-\alpha /2-\epsilon )-\Phi^{-1}(1-\alpha /2)<0$. Accordingly, it follows by Lemma 2 that

$$F_{W}\left\{\Phi^{-1}(1-\alpha /2+\epsilon )\right\}⟶1\;\text{and}\;F_{W}\left\{\Phi^{-1}(1-\alpha /2-\epsilon )\right\}⟶0$$

in probability as $n_{T}\to \infty$, and so

$$\Phi \left(\frac{b_{n_{T}}^{\alpha }-{\tilde{x}}^{⊤}\theta_{T}}{\sigma }\right)=\Phi (W)⟶1-\alpha /2$$

in probability as $n_{T}\to \infty$. A similar argument shows that

$$\Phi \left(\frac{a_{n_{T}}^{\alpha }-{\tilde{x}}^{⊤}\theta_{T}}{\sigma }\right)⟶\alpha /2,$$

in probability as $n_{T}\to \infty$, concluding the proof. ■

## Appendix B. Bounding Continuous Integral

Recall the posterior distribution of the calibration parameters for the continuous response setting,

$$\pi \left(\delta ,\gamma ,\sigma |y_{T,1},\ldots ,y_{T,n_{T}},{\hat{\theta }}_{S}\right)\;=\pi \left(\delta ,\gamma ,\sigma \right)\cdot \prod_{i=1}^{n_{T}}\int_{R}\frac{\text{Cauchy}\left(\beta_{i}|\delta ,\gamma \right)}{\left|f\left({\hat{\theta }}_{S},x_{T,i}\right)\right|}\cdot 𝒩\left\{\beta_{i}|\frac{y_{T,i}}{f\left({\hat{\theta }}_{S},x_{T,i}\right)},\frac{\sigma^{2}}{f{\left({\hat{\theta }}_{S},x_{T,i}\right)}^{2}}\right\}d\beta_{i}.$$

Calculating this posterior requires the evaluation of $n_{T}$ integrals over R. For computational efficiency, we estimate the posterior by integrating over closed intervals. The incurred numerical error can be tuned to be lower than computer precision.

Performing the substitution $u_{i}=\left\{\beta_{i}-y_{T,i}/f\left({\hat{\theta }}_{S},x_{T,i}\right)\right\}/\left\{\sigma /\left|f\left({\hat{\theta }}_{S},x_{T,i}\right)\right|\right\}$ re-expresses the ith integral as

$$\int_{\mathbb{R}}\frac{𝒩\left(u_{i}|0,1\right)}{\sigma }\cdot \text{Cauchy}\left[u_{i}|\frac{\left|f\left({\hat{\theta }}_{S},x_{T,i}\right)\right|}{\sigma }\left\{\delta -\frac{y_{T,i}}{f\left({\hat{\theta }}_{S},x_{T,i}\right)}\right\},\frac{\left|f\left({\hat{\theta }}_{S},x_{T,i}\right)\right|\gamma }{\sigma }\right]du_{i}\leq \frac{1}{\sigma }\left(\int_{s_{1}}^{s_{2}}𝒩\left(u_{i}|0,1\right)\cdot \text{Cauchy}\left[u_{i}|\frac{\left|f\left({\hat{\theta }}_{S},x_{T,i}\right)\right|}{\sigma }\left\{\delta -\frac{y_{T,i}}{f\left({\hat{\theta }}_{S},x_{T,i}\right)}\right\},\frac{\left|f\left({\hat{\theta }}_{S},x_{T,i}\right)\right|\gamma }{\sigma }\right]du_{i}+\phi \left(s_{1}\right)+\phi \left(s_{2}\right)\right),$$

for any $s_{1}$ and $s_{2}$ satisfying $s_{1}<0<s_{2}$, where ϕ(·) is the standard Gaussian density function. Then choose $s_{1}$ and $s_{2}$ so that $\phi \left(s_{1}\right)+\phi \left(s_{2}\right)$ is as small as desired. For example, we set $s_{1}=-39$ and $s_{2}=39$, giving $\phi \left(s_{1}\right)$ and $\phi \left(s_{2}\right)$ numerically equal to zero in the base Julia software (for comparison, $\phi (38)=1.097\times 10^{-314}$).

## Appendix C. MCMC Implementation Details

Sections 4.2 and 5.2 detail the procedure for sampling from the posterior predictive distribution of a new observation. RECaST first estimates the joint posterior density of the re-calibration parameters (δ,γ,σ) in the linear model and (δ,γ) in the logistic model. We specify disperse priors δ~𝒩(1,400),log(γ)~𝒩(0,9), and in the continuous setting $\text{log}\;\left(\sigma^{2}\right)\sim 𝒩(0,9)$. We run the Metropolis-Hastings estimation algorithm of the posterior distribution for 100,000 iterations with the initial 20,000 iterations used as a burn-in period to tune the proposal variance. The parameters from the final 50,000 iterations are used as the posterior distribution. Finally, $n_{\text{post}}=300$ equally spaced triplets/pairs of this distribution are taken as a posterior sample to be used in the posterior predictive estimation, which we denote by ${\left\{\delta_{i},\gamma_{i},\sigma_{i}\right\}}_{i=1}^{300}$ and ${\left\{\delta_{i},\gamma_{i}\right\}}_{i=1}^{300}$ in the linear and logistic models respectively. For each triplet/pair, a sample of $n_{\beta }=300\beta$’s are taken from the Cauchy distribution, each used to generate $n_{Y}=300$ samples from the posterior predictive distribution. This gives 300 × 300 × 300 = 27, 000, 000 posterior predictive observations for each out-of-sample test point, $\left(Y_{T,\text{test}},{\tilde{x}}_{T}\right)$.

## Appendix D. Neural Network Training Procedure

The following procedure is used to train all neural networks considered: the source DNN, the DNN trained only on target data, and the Unfreeze DNN.

We initialize the weights using Xavier initialization (Glorot and Bengio, 2010). The network is trained for 2500 epochs using the ADAM optimizer and an MSE loss. A portion of the training data is set aside as an out-of-sample calibration set during training. At each epoch, the training and calibration loss are tracked. The final parameterization used is taken from the epoch with the lowest calibration loss to avoid overfitting.

The candidate architectures ranged from networks with 316 parameters to 11,641 parameters with varied number of layers, layer sizes, activation functions, and dropout proportions. The architecture described below was chosen as it had the best test set AUC on the eICU data of all considered architectures. We use a two layer neural network with layer sizes $\ell_{1}=(p,25)$ and $\ell_{2}=(25, 1)$. These layers are connected with a Rectified Linear Unit (ReLU) activation function. In the binary response setting, the output of $\ell_{2}$ is converted to a probability through a softmax activation function. For consistency, this architecture is also used for the simulated data analysis in Section 6.

The source neural network for RECaST learns parameters in both layers using only source data. The DNN network learns parameters in both layers using only the target data. The Unfreeze DNN network learns parameters in both layers first using only the source data. Then, the target data are processed through the same neural network, re-training parameters in the second layer and leaving the first layer unchanged from the values learned on the source data set.

## Appendix F. Additional Robustness Results

Here, we consider the case where $\theta_{S}$ and $\theta_{T}$ are orthogonal. We take $\theta_{S}$ to be the same p=50 feature vector as in Section 6.1 and take $\theta_{T}$ to be a vector in the null space of $\theta_{S}$. The data are otherwise generated following Section 6.1 from a linear or logistic regression with a fixed source sample size of $n_{S}=1000$ and a varying target sample size of $n_{T}\in \{20, 40, 60, 100, 250\}$.

In the continuous outcome case, we compare RECaST to the DNN, Unfreeze DNN, TransRF and glmtrans approaches. In the binary outcome case, we compare RECaST to the target-only DNN, Unfreeze DNN, TransRF, glmtrans, WDGRL and Wiens methods. Table 14 shows the predictive performance of RECaST for the orthogonally misaligned source and target setting with a continuous response.

**Table 12:** Empirical coverage (standard error) at the 95% nominal level for a continuous response, averaged over 300 source and target data sets when the when the target model parameters are generated as $\theta_{T}=\theta_{S}+\epsilon$ with $\epsilon \sim {𝒩}_{p\times 10+10\times 1}(0,0.025I)$. The out-of-sample test sets each contain 250 observations. All reported values are multiplied by 100.

$n_{T}$	RECaST LM	RECaST DNN
250	100(0.4)	100(0.28)
100	100(0.33)	100(0.33)
60	100(0.35)	100(0.32)
40	100(0.29)	100(0.32)
20	100(0.37)	100(0.37)

**Table 13:** Empirical coverage (standard error) at the 95% nominal level for a continuous response, averaged over 300 source and target data sets when the source and target neural network weight matrices are orthogonal. The out-of-sample test sets each contain 250 observations. All reported values are multiplied by 100.

$n_{T}$	RECaST LM	RECaST DNN
250	100(0.53)	100(0.24)
100	100(0.42)	100(0.68)
60	100(0.36)	100(0.3)
40	100(0.3)	100(0.93)
20	100(0.26)	100(0.25)

**Table 14:** Out of sample RMSE (standard error) averaged over 300 source and target data sets when the source data generating parameters are orthogonal to the target data generating parameters. The out-of-sample test sets each contain 250 observations. All reported values are multiplied by 100.

$n_{T}$	LM	DNN	RECaST LM	RECaST DNN	Unfreeze DNN	TransRF	glmtrans	MTL FO	MTL MoM
250	0.56(0.03)	0.85(0.061)	1.1(0.051)	1.1(0.051)	0.92(0.082)	0.63(0.066)	0.54(0.025)	0.6(0.03)	0.6(0.03)
100	0.71(0.06)	1.1(0.12)	1.1(0.050)	1.1(0.051)	1.1(0.19)	0.97(1.1)	0.55(0.031)	0.76(0.06)	0.76(0.06)
60	1.3(0.28)	1.3(0.13)	1.1(0.05)	1.1(0.051)	1.2(0.28)	1.8(2.0)	0.57(0.043)	1.3(0.26)	1.3(0.26)
40	1.2(0.22)	1.3(0.13)	1.1(0.056)	1.1(0.056)	1.5(0.40)	3.0(6.2)	0.6(0.066)	3.2(0.71)	1.4(0.29)
20	1.0(0.07)	1.4(0.14)	1.2(0.078)	1.2(0.073)	1.9(0.59)	-	0.66(0.08)	5.5(0.81)	1.0(0.07)

When the target data are plentiful $\left(n_{T}=100, 250\right)$ the RMSE for the LM built solely on the target data outperforms the RECaST methods. This aligns with previous results where there is a large amount of data and a large discrepancy between the source and target (i.e., when transfer learning is not appropriate). As the number of target data points decreases, RECaST outperforms target-only DNN. These results further demonstrate the robustness of RECaST to negative transfer. Notice that glmtrans is also robust; in each of these scenarios glmtrans opted to not use the source data. The MTL MoM method also provides good predictive performance for all sample sizes without requiring access to the source data. Similar to the previous robustness tests, Table 15 shows that RECaST provides conservative predictive intervals resulting in over-coverage at the 95% level.

**Table 15:** Empirical coverage (standard error) at the 95% nominal level, averaged over 300 source and target data sets when the source data generating parameters are orthogonal to the target data generating parameters. The out-of-sample test sets each contain 250 observations. All reported values are multiplied by 100.

$n_{T}$	RECaST LM	RECaST DNN
250	100(0)	100(0)
100	100(0)	100(0)
60	100(0)	100(0)
40	100(0)	100(0)
20	100(0.024)	100(0.024)

For a binary response, Table 16 shows the difficulty of this problems. All methods have very low AUCs, including the target-only DNN. For large sample sizes, glmtrans performs relatively well, again due to its ability to ignore the source data entirely and because the model matches the data generating mechanism. Table 17 shows that all methods have predictive coverages with very high standard errors, again displaying the difficulty of this problem.

Next we consider a setting in which the source feature space is a subset of the target feature space: ${𝒳}_{S}⊊{𝒳}_{T}$. We assign 12 features to the true target data $X_{T}$ but only 9 features to the true source data $X_{S}$. The parameters are generated as $\theta_{T}=(-a,b)$ where $a,b\in R^{6}$ have components independently sampled from Uniform(0.75, 5), and $\theta_{S}=\left[\theta_{T,1},\ldots ,\theta_{T,9}\right]$, the first nine components of $\theta_{T}$. The responses, $Y_{S}$ and $Y_{T}$, are generated via linear or logistic regression with their respective feature vectors.

Table 18 shows that for a continuous response, every method has similar predictive performance when the target sample size is large. As the target sample size decreases, RECaST and glmtrans have the best performance, maintaining a stable RMSE value and outperforming the target-only DNN. This shows that RECaST is robust to negative transfer in this setting.

**Table 16:** Out-of-sample AUC (standard error) averaged over 300 source and target data sets when the source and target model parameter vectors are orthogonal. The out-of-sample test sets each contain 250 observations. All reported values are multiplied by 100.

$n_{T}$	DNN	RECaST GLM	RECaST DNN	Wiens	Unfreeze DNN	TransRF	glmtrans	WDGRL
250	59(5.6)	51(3.1)	50(3.7)	49(3.6)	55(4.7)	57(9.1)	72(3.5)	49(3.7)
100	54(5.3)	50(3.5)	49(3.4)	45(3.7)	53(4.7)	37(14)	69(7.1)	50(3.5)
60	52(5.3)	50(3.8)	50(4.2)	43(3.4)	51(4.7)	25(16)	61(10)	48(3.6)
40	52(4.9)	50(3.5)	50(3.8)	42(2.9)	52(4)	23(16)	59(9.1)	49(4)
20	52(4.8)	50(3.5)	50(3.4)	41(4.1)	51(4.5)	-	56(9.5)	-

**Table 17:** Empirical coverage (standard error) at the 75% nominal level, averaged over 300 source and target data sets when the source and target model parameter vectors are orthogonal. The out-of-sample test sets each contain 250 observations. All reported values are multiplied by 100.

$n_{T}$	DNN	RECaST GLM	RECaST DNN	Wiens	Unfreeze DNN	TransRF	glmtrans	WDGRL
250	59(11)	100(0)	100(0)	53(10)	55(10)	61(13)	81(11)	52(17)
100	55(11)	83(29)	100(0)	52(11)	52(11)	54(13)	77(15)	47(18)
60	50(11)	89(19)	51(10)	48(11)	49(12)	52(15)	74(19)	47(17)
40	52(11)	45(16)	77(27)	47(11)	52(11)	52(27)	67(19)	48(10)
20	51(11)	60(27)	57(25)	51(12)	51(14)	-	59(13)	-

Table 19 shows RECaST again provides conservative predictive intervals at the 95% level. We see similar results for a binary response outcome in Table 20. Both RECaST methods have stable AUCs as the target sample size decreases, outperforming the target-only DNN and the other transfer learning methods. WDGRL and glmtrans also perform well for larger sample sizes, but both require access to the source data while training. Table 21 shows that the RECaST and Wiens methods again provide conservative predictive coverage for all target sample sizes. WDGRL and glmtrans under-cover in some scenarios.

**Table 18:** The reported values are: average out-of-sample RMSE (standard deviation). These summaries are over all 300 different source and target data sets for each target sample size when the target data had more features than the source.

$n_{T}$	LM	DNN	RECaST LM	RECaST DNN	Unfreeze DNN	TransRF	glmtrans	MTL FO	MTL MoM
250	5.5(1.3)	5.8(1.4)	5.4(1.3)	5.4(1.3)	5.52(1.36)	6.5(1.5)	5.3(1.2)	6.3(1.2)	6.3(1.2)
100	5.7(1.3)	6.3(1.5)	5.4(1.3)	5.4(1.3)	5.68(1.35)	11.0(14.0)	5.4(1.2)	6.5(1.2)	6.5(1.2)
60	5.8(1.4)	6.8(1.7)	5.4(1.3)	5.4(1.3)	5.94(1.53)	14.0(14.0)	5.3(1.2)	6.7(1.3)	6.7(1.3)
40	6.2(1.5)	7.2(1.8)	5.4(1.4)	5.4(1.4)	6.06(1.76)	30.0(43.0)	5.4(1.3)	6.9(1.4)	6.9(1.4)
20	7.3(2.1)	8.2(1.9)	5.5(1.3)	5.5(1.4)	6.63(1.93)	-	5.6(1.5)	8.1(1.9)	8.1(1.9)

**Table 19:** Empirical coverage (standard error) at the 95% nominal level, averaged over 300 source and target data sets when the target data had more features than the source. The out-of-sample test sets each contain 250 observations. All reported values are multiplied by 100.

$n_{T}$	RECaST LM	RECaST DNN
250	100(0)	100(0)
100	100(0)	100(0)
60	100(0)	100(0)
40	100(0)	100(0)
20	100(0.022)	100(0.2)

**Table 20:** Out-of-sample AUC (standard error) averaged over 300 source and target data sets when the target data had more features than the source. The out-of-sample test sets each contain 250 observations. All reported values are multiplied by 100.

$n_{T}$	DNN	RECaST GLM	RECaST DNN	Wiens	Unfreeze DNN	TransRF	glmtrans	WDGRL
250	86(5.3)	89(4.5)	89(4.5)	74(7.9)	89(3.9)	60(16)	90(5.9)	89(6.5)
100	83(10)	89(4.4)	89(4.3)	75 (8.0)	84(10)	46(18)	88(4.1)	89(4.2)
60	75(13)	89(4.2)	89(4)	74(7.2)	79(12)	38(21)	86(3.8)	90(3.4)
40	73(11)	89(4.3)	89(4.3)	73(7.3)	78(14)	32(12)	82(9.2)	89(4.0)
20	75(7.9)	88(4.5)	88(4.3)	74(6.6)	81(9.6)	-	69(13)	-

**Table 21:** Empirical coverage (standard error) at the 75% nominal level, averaged over 300 source and target data sets when the target data had more features than the source. The out-of-sample test sets each contain 250 observations. All reported values are multiplied by 100.

$n_{T}$	DNN	RECaST GLM	RECaST DNN	Wiens	Unfreeze DNN	TransRF	glmtrans	WDGRL
250	70(14)	100(0)	98(1.6)	87(11)	75(13)	73(14)	71(12)	63(15)
100	65(7.6)	96(0)	95(7.3)	85(8.9)	73(12)	64(15)	72(11)	64(13)
60	67(16)	89(6.5)	90(12)	86(8.2)	70(14)	58(22)	79(13)	69(9.9)
40	60(11)	86(16)	88(11)	86(8.2)	72(13)	53(17)	74(15)	63(17)
20	59(12)	82(17)	80(19)	86(9.8)	67(19)	-	70(16)	-

## Appendix H. eICU Feature Descriptions

**Table 22:** Descriptions of the features from the eICU Collaborative Research Database used in the shock data analysis.

Variable	Description
Age	Age in years
Gender	Gender as either Male, Female, Unknown or Other
Ethnicity	Ethnicity as either Asian, Caucasian, African American, Native American, Hispanic or Other/Unknown
Weight	Weight upon admission
Temperature	Worst temperature measured from a midpoint of 38°C
White blood cell count	Worst white blood cell count from a midpoint of 11,500 white blood cells per microliter
Respiratory rate	Worst respiratory rate from a midpoint of 19 breaths per minute
Heart rate	Worst heart rate from a midpoint of 75 beats per minute
Hematocrit level	Worst hematocrit from a midpoint of 45.5%
Creatinine level	Worst serum creatinine from a midpoint of 1.0 milligrams per deciliter
Glucose level	Worst glucose from a midpoint of 130 milligrams per deciliter
Oxygen saturation	Oxygen saturation in the blood measured by a pulse oximeter
Dialysis	An indicator reporting if the patient is on dialysis
Intubated	An indicator reporting if the patient was intubated during the worst measurement of their arterial blood gas
Ventilated	Binary an indicator reporting if the patient was ventilated during the measurement worst respiratory rate
Eye	Eye score ranging from 1 to 4 on the Glasgow Coma Scale
Motor	Motor score ranging from 1 to 6 on the Glasgow Coma Scale
Verbal	Verbal score ranging from 1 to 3 on the Glasgow Coma Scale
